# Mechanical Response of FeNiCrCoAl High-Entropy Alloys at the Nanoscale: Predictions from Molecular Dynamics

**DOI:** 10.3390/nano15090652

**Published:** 2025-04-25

**Authors:** Ernesto Amaro, Joaly Delgado-Alvarez, Jairo Andrés Martínez-Uribe, Sergio Mejía-Rosales

**Affiliations:** 1 Facultad de Ciencias Físico-Matemáticas, Universidad Autónoma de Nuevo León, San Nicolás de los Garza 66455, NL, Mexico; angel.amarodln@uanl.edu.mx (E.A.); joaly.delgadoa@uanl.edu.mx (J.D.-A.); 2 Centro de Investigación en Ciencias Físico-Matemáticas (CICFIM), Facultad de Ciencias Físico-Matemáticas, Universidad Autónoma de Nuevo León, San Nicolás de los Garza 66455, NL, Mexico; andres.martinezurb@uanl.edu.mx

**Keywords:** FeNiCrCoAl high-entropy alloys, dislocation dynamics, stress–strain behavior, simulated STEM micrographs, mechanical properties at the nanoscale

## Abstract

The mechanical response of high-entropy alloys (HEAs), specifically the FeNiCrCoAl HEA, was studied at both bulk and nanoparticle scales using molecular dynamics simulations. These simulations were performed using the LAMMPS software with an Embedded Atom Method (EAM) potential. The results show that Bulk HEAs exhibited enhanced hardening and plasticity, while in nanoparticles, distinct deformation patterns were observed, including nanotwin formation, V-shaped stacking fault planes, and intermittent dislocation activity due to free surface effects. The crystallographic orientation with respect to the compression significantly affected the deformation mechanisms, with the [100] direction favoring progressive hardening, while the [110] and [111] directions exhibited different stacking fault and dislocation dynamics. A detailed analysis using von Mises stress and dislocation analysis provided insights into the effects of scale on mechanical properties.

## 1. Introduction

Interest in the development of novel high-entropy alloys (HEAs) has been growing steadily, which is primarily due to their exceptional mechanical properties, including high strength, hardness, and corrosion resistance; the main reason for this growing interest is that in general, HEAs exhibit unique mechanical properties, such as high strength, high hardness, and corrosion resistance [1,2]. In particular, research has strongly focused on understanding the phase stability, mechanical response, and microstructural evolution of these alloys, especially at high temperatures, where stability may be critical for the use of these materials in specific applications [3]. These alloys, which consist of at least three principal metal elements, each with atomic proportions from 5 to 25 atomic percentage, offer promising characteristics for advanced engineering applications. A key area of research has focused on understanding their phase stability, mechanical performance, and microstructural evolution, particularly at elevated temperatures, where material stability is crucial for specific applications [4]. One significant finding in this field is that FeMnCrNiAl alloys exhibit enhanced strength, with aluminum concentrations up to 10 at.%. However, increasing the aluminum content beyond this level does not yield further strength improvements [3,5]. Related to this, the role of microstructural features, such as deformation twins and lamellar structures, has been pointed out as critical for improving the mechanical properties of refractory HEAs. These insights, as discussed by Zhang et al. [6], underscore the importance of understanding the atomic-level mechanisms that influence the material’s behavior. In particular, aluminum addition in the Al_*x*_CoCrFeNi system promotes a transition to a body-centered cubic (BCC) structure, leading to significant increases in hardness through spinodal decomposition mechanisms [7]. Guided by these findings, this study employs molecular dynamics simulations to investigate the nanoscale mechanical behavior of FeNiCrCoAl HEAs, with a focus on how aluminum alters atomic-scale local arrangement and, hence, the mechanical response of the material. While previous research on HEAs has largely centered on their bulk properties, relatively smaller attention has been paid to the mechanical response of these systems as nanostructures. While this scarcity of information is understandable given the practical difficulty of obtaining precise and quantitative measurements at the nano scale, the need of understanding confinement, surface effects, and limited defect dynamics cannot be understated. In consonance with this, the main objective of this work is to investigate and compare the bulk mechanical responses against nanoparticle FeNiCrCoAl alloys subjected to external compression by the use of molecular dynamics simulations and several analysis tools. In particular, we pay attention to the role of aluminum in altering atomic-scale mechanical responses. By examining the stress–strain behavior, dislocation activity, and microstructural changes, this study aims to shed light on the effects of integration at the nano scale on the mechanical properties of Al_*x*_CoCrFeNi and possibly other HEAs. Our study presents an approach based on molecular dynamics simulations to examine how Al content influences the compressive behavior of FeNiCrCoAl HEAs. By comparing bulk and nanoparticle responses across different crystallographic orientations, we reveal atomic-level mechanisms of strength and twinning in these systems, and through the simulation of high-resolution STEM imaging, we offer a fair comparison between the simulation and experimental observations.

## 2. Methods

### 2.1. Interaction Model

Molecular dynamics simulations were conducted using the LAMMPS software, release of 2 August 2023, employing an Embedded Atom Method (EAM) potential, as described by Farkas and Caro [8]. While Farkas and Caro state that the implementation of this interaction model aims to explain trends rather than precise quantitative predictions, its ability to accurately capture lattice distortions, short-range ordering effects, and stacking fault energy variations makes it particularly useful for studying mechanical responses under applied stress [9]. Ref. [10] , for example, used this interaction model to investigate the nanoscale scratch behavior of an FeNiCrCoAl HEA, using molecular dynamics (MDs) simulations; they found that the frictional and wear characteristics of FeNiCrCoAl are significantly influenced by the temperature and Fe concentration. In particular, the potential of Farkas and Caro appropriately describes how the addition of Al induces lattice strain, and it reproduces the experimentally observed influence of the Al content on the stacking fault energy. While the precision in the calculation of specific critical quantities is limited, on the other hand, the potential may be a valuable tool to better understand the role of mixing, defects dynamics, and phase coexistence, not only in this specific alloy but in general in FCC multimetallic alloys. Taking all this into consideration, and in order to verify the predictability features of the potential, a set of MD runs was made to calculate the elastic moduli of monometallic blocks of 15 × 15 × 15 unit cells; the results were compared against accepted experimental values in Table 1. As it could be expected from an interaction potential designed to reproduce more trends than exact values [8], there are differences in comparison with the experimental and DFT-obtained values, but for most of the cases, these differences are not larger than 20%.

### 2.2. Building up the Systems

**Figure 1:**
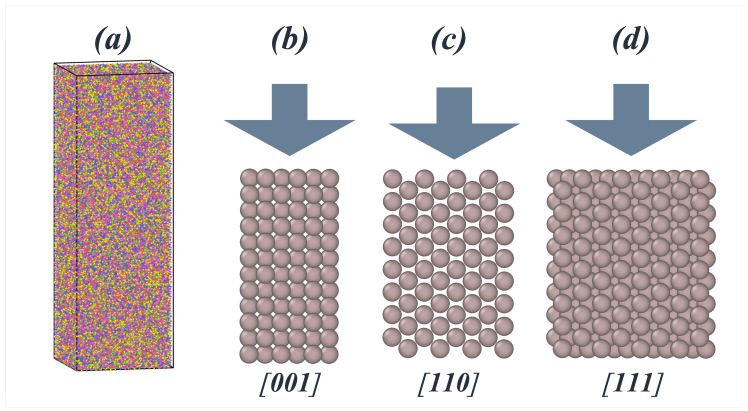
(**a**) Atomistic representation of the simulated systems; each color represents a different element. (**b**–**d**) Representations of the atomistic ordering with respect to direction of compression: (**b**) [100]; (**c**) [110]; (**d**) [111].

In this work, two distinct systems were modeled: the alloys as a bulk system and as an isolated nanoparticle. The bulk system comprised a 20 × 20 × 60 supercell in a face-centered cubic (FCC) structure made of approximately 96 × 103 atoms and covering a volume of 1039 nm^3^. Both bulk and nanoparticle systems were prepared such that Fe, Ni, Cr, and Co appeared randomly in the volume of the system at approximately the same proportions, while Al was present at a comparatively lower concentration. Four Al compositions were considered: 0%, 2.4%, 7.2%, and 11.2%. Since hardness, elastic modulus, and yield strength are likely to depend on the indentation direction relative to the different crystallographic directions due to both atomistic arrangement and packing, we built models where the indentation was performed parallel to one of these three crystallographic directions: [001], [110], or [111]. Figure 1 shows a representation of each of these three cases, along with an atomistic representation of the HEA’s volume. For the model of isolated nanoparticle, we used the same sizes, atomic distributions, arrangements, and orientations as for bulk, but the periodicity of the system was avoided by defining the simulation box such that its borders parallel to the compression were far away from the surface of the volume of the HEA.

### 2.3. Simulation Procedure

The simulations were performed using the Large-scale Atomic/Molecular Massively Parallel Simulator (LAMMPS), which is the well-known open-source molecular dynamics code [14]. Each system was subjected to energy minimization using the conjugate gradient method to achieve a stable initial configuration. Then, the material was equilibrated at 300 K using an MD run in the NVT ensemble, followed by temperature ramping from 300 K to 1500 K and back under the NPT ensemble to prepare the structures for mechanical testing. Afterwards, each system was subjected to compression along the *z* axis at a strain rate of $1\times 10^{9}$ $s^{-1}$ under the NPT ensemble. While this strain rate is far from the typical experimental conditions, it is still low enough to capture meaningful atomic-level behavior, and it actually promotes the appearance of atomistic-level responses that indeed occur in real systems under compression [15]. It is common to work in molecular simulations at strain rates of the order of $1\times 10^{9}$ $s^{-1}$, which is a rate low enough to avoid amorphous-disordered deformation and constrained free vibrations [16]. For the bulk systems, compression was attained by deforming the simulation box in the *z* direction, while for the nanoparticles, two nonatomistic indenters parallel to *z* were defined at opposite ends of the nanoparticle, with one of them approaching the other at the strain rate already mentioned; these planes interact with the nearest atoms trough the exertion of a quadratic force [14], with the simulations carried out under the NVT ensemble. As usual, the simulation time step was set to 1 fs, and temperature, energy, and pressure were monitored during the whole process.

### 2.4. Analysis

We used several analysis tools, as implemented in the most current version of the OVITO visualization and analysis software [17]. The Dislocation Extraction Algorithm (DXA) [18] was used to identify and track dislocations during compression and to quantify the evolution of dislocation density as the system evolved. W Orientation Analysis [19] was performed to study changes in the crystallographic orientation of the domains and in particular to identify the appearance of nanotwins in the structure during plastic deformation. The identification of different local structural environments was usually made using common neighbor analysis (CNA) [20], and DXA was also used for this purpose in some instances. For the investigation of local stress, von Mises stress fields were computed at each timestep to follow the progression of both elastic and plastic deformation [21] using the methodology already described by one of the coauthors in a previous work on Cu nanoparticles [22]. In order to enable the comparison against experimental observations, in several instances we obtained simulated high-angle annular dark-field scanning transmission electron micrographs (HAADF-STEMs) using the Prismatic v2.0 software [23]. We generated the simulated HAADF-STEMs using the multislice method as implemented in the Prismatic program; for this, we used a set of microscope parameters facilitated for an experienced electron microscopist in order to obtain sub-Angstrom resolution and Z-contrast: Acceleration voltage: 200 kV; Probe Semi angle = 10 mrad; C3 aberration = 0; C5 aberration = −0.005 cm; detector semiangle range: 45–60 mrad; probe step = 0.25 Å. Thermal effects were considered by calculating the average of the RMS atomic displacement along an NVT MD trajectory at 300K. It is worth remarking here that, given the relatively large size of the simulated systems, it was possible to generate HAADF-STEM simulated images only for a subset of particular orientations, since the simulation algorithm makes extensive use of RAM memory to allocate the data, and some orientations required an amount of memory that surpassed the capacity of our computer. The size of the systems also made compulsory the use of a high-capacity graphics processing unit (GPU) for the parallel computation of the interaction of the electron beam with the sample; for this, we made use of a NVIDIA GeForce RTX 4090 (24 GB VRAM) GPU. The molecular dynamics simulations were performed also in this GPU, altogether with NVIDIA RTX 4070 Ti (12 GB VRAM), RTX 3060 (8 GB VRAM), and Quadro RTX 4000 (8 GB VRAM) GPUs, all running with CUDA version 12.2 [24] .

## 3. Results

### 3.1. Elastic Regime

**Figure 2:**
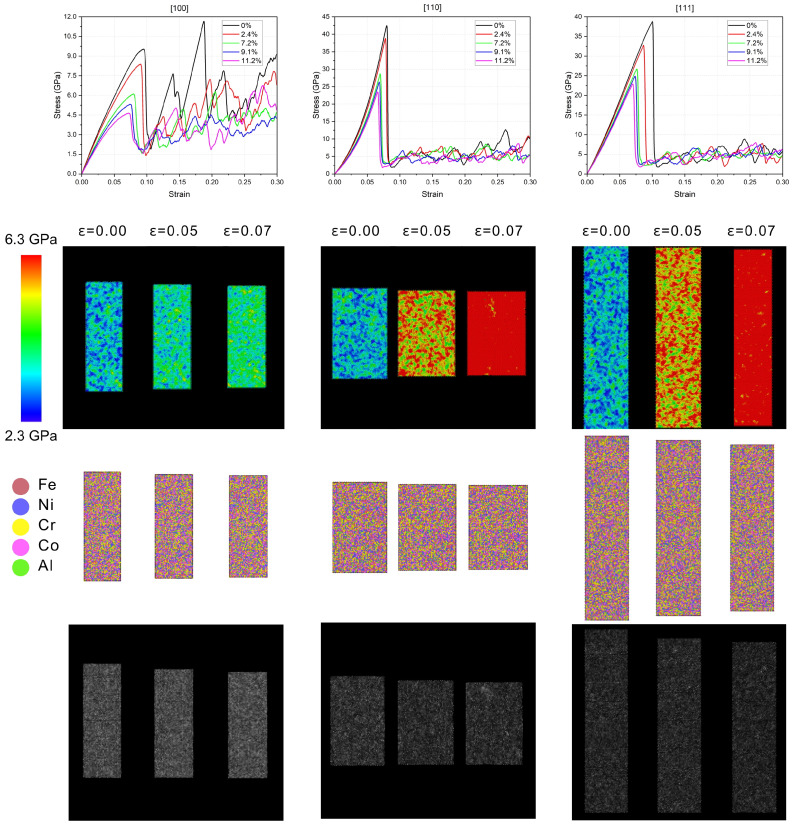
First row: Compressive stress vs. strain curves for the several values of Al content. Second row: Distribution of von Mises stress at three values of strain, at the elastic regime, for systems with 7.2 at. % of Al; left: compression perpendicular to [100]; center: compression perpendicular to [110]; right: compression perpendicular to [100]. Third row: Atomistic representation of the same systems of the second row. Fourth row: Simulated HAAD-STEMs of the same systems of the second row.

Figure 2 shows characteristic stress–strain curves obtained at the three crystallographic directions considered in this study. From the curves, it is worth noting that, irrespective of either Al content or crystallographic direction, the calculated values of compressive strength reached values considerably larger than what is usually measured either in stainless steels or even in nickel-based superalloys, which for Fe-Ni-Cr systems lie in the order of units of GPa [25]. While these unreal results can at least partially be related to the Hall–Petch relationship—since the starting configuration is a monocrystal—it is more likely that the strongest effect is due to the limitations of the interaction model; nevertheless, even though this discrepancy highlights the necessity for careful parameterization of the interaction potentials and validation against experiments, we believe that the overall qualitative features and the differences in the mechanical behavior between the simulated systems allow for a valid interpretation for understanding the effect of composition in this kind of high-entropy alloy.

As can be noted clearly from the graphs on the upper row of Figure 2, while the elastic regime is well defined in all of the systems, in those subjected to compression in the [100] direction, there was a slight tendency to softening as the strain was increased, which is evidenced by the lowering of the slope of the stress–strain curve in contrast to compression in the [100] direction, where the opposite occurred. These differences in the behavior of the change of slope can be attributed to the differences between the packing of [100], [110], and [111] planes; in the relatively open structure of the [100] planes, the initially aligned bonds experienced a progressive misalignment under the compression force, which resulted in a decreasing slope of the stress–strain curve and thus the softening behavior as the strain increased. In the [110] planes, with higher atomic packing density than the [100] planes, repulsion dominated as the strain was increased, resulting in slight hardening behavior. In the more densely packed arrangement of the [111] planes, the stress distribution remained more uniform under strain, and the system maintained a more closely linear elastic response.

As expected in fcc structures, the anisotropy of Young’s modulus followed the trend $E_{\left[111\right]}>E_{\left[110\right]}>E_{\left[100\right]}$, and, in agreement with this, the local distribution of stress followed the same trend, as can be noted in the representations of the von Mises stress distributions shown in Figure 2 in the lower row. In the figure, the distribution of von Mises stress is shown at three values of strain, while based on the system (7.2% of Al) and from the color scale we can note that, in the same range of strain, compression parallel to the [100] planes generated a local stress lower than in the other two directions.

**Figure 3:**
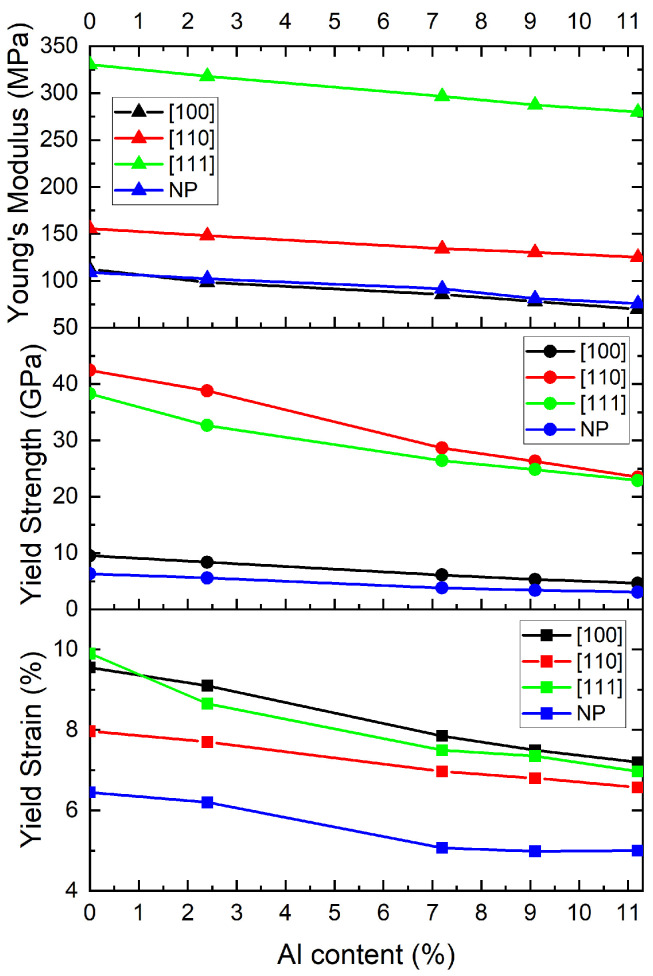
Young’s modulus (**top**), yield strength (**middle**), and yield strain (**down**) as function of Al content for the three compression directions of bulk and for the HEA nanoparticle.

Figure 3 summarizes the effect that the Al content has on the mechanical response in the elastic regime. Unexpectedly, both the yield strength and yield strain decreased as the Al content was increased, irrespective of the direction of the compression and even when the HEA formed a nanostructure, which is marked as NP in the Figure. Similar trends have been reported by other authors [26,27,28]. We believe that, since the Al content is small, its effect in solid solution strengthening is practically nonexistent in comparison to the effect of the other elements in the creation of lattice distortions that hinder dislocation movement and explain at least partially the high values of the yield strength. Related to this, our observation that increasing the Al content lowered the yield strength and yield strain in our HEA models likely stems from the uniform distribution of Al in our models; experimental HEAs often exhibit chemical short-range order (CSRO), where elements like Al preferentially cluster or form local chemical patterns that enhance mechanical strength. For example, Li et al. and Cao found that CSRO increases stacking fault energies and impedes dislocation motion via nanoscale detrapment mechanisms, effectively increasing strength and delaying plastic deformation [29,30]. Other studies show that CSRO structures—especially Ni- or Co–Cr-based structures—create dislocation pinning sites and promote complex dislocation interactions, which leads to higher resistance to nucleation and enhanced work hardening [31,32]. Woodgate et al. give theoretical support for the appearance of $L1_{2}$ ordering driven by the presence of Al in an Fe-Mn-Cr-Ni-Al alloy, where Al takes specific sites in a simple cubic lattice [33]. The lack of CSRO in our models may at least partially explain the difference in yield properties against what is expected experimentally; further analysis is being conducted to try to better understand the effect of Al at these low percentages.

### 3.2. Plastic Regime

**Figure 4:**
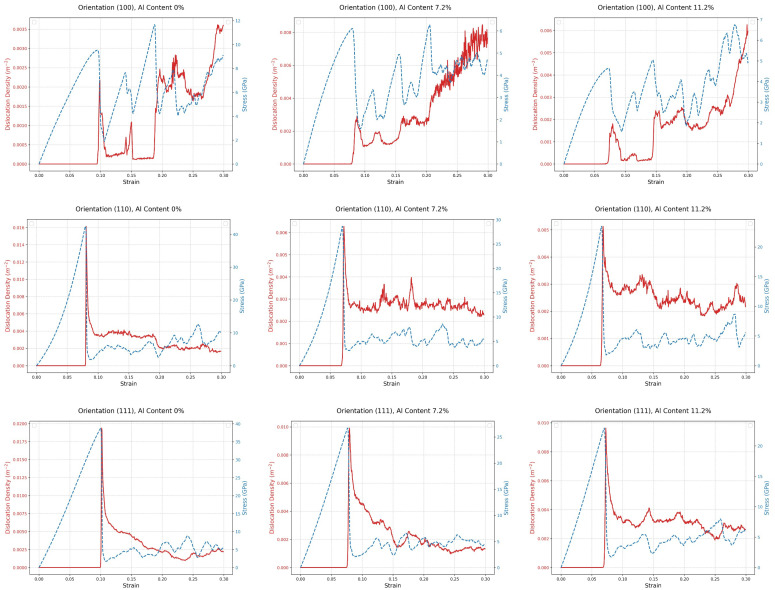
Compressive stress and dislocation density vs. strain. Top: [100] direction. Middle: [110] direction. Right: [111] direction. Left: 0% Al. Center: 7.2% Al. Right: 11.2% Al.

Once the compression process reaches the plastic regime, the dynamics of dislocations become strongly dependent on the compression direction. This is directly visualized in Figure 4, where we have plotted in a same plane both the compressive stress and dislocation density vs. strain for each of the orientations and three of the compositions (0%, 7.2%, and 11.2% Al).

As can be noted by the behavior of the dislocation density plots, the dynamics of the formation of dislocations were more sensitive to the direction of compression than to Al content. In particular, when the compressive force pointed perpendicular to the {100} planes, irrespective of the content of aluminum, at high values of strain (ϵ⪆0.2), the material started to experience hardening, and this coincided with an increase in the density of the dislocations. Inspection of the dynamical trajectories for the dislocations shows that at around this range of values of ϵ, the high density of dislocations produced dislocations that started to interact with each other, leading to entanglement and obstruction of movement. As a consequence, further deformation becomes more difficult, hence resulting in the increase in the value of the compression stress.

In general, as can be noted in Figure 4, there was a nonmonotonic evolution of dislocation density characterized by a sharp increase at the start of the plastic regime and followed by fluctuations at higher strain levels. While the dislocation density usually increases continuously with strain, such fluctuations are not uncommon in molecular dynamics simulations and can be attributed to a combination of physical and numerical factors. In our simulations, periodicity was applied in the *x* and *y* directions, but not in the *z* direction, which is the direction of applied compressive strain; this setup allows dislocations to evolve and escape along the loading direction without artificial constraints, thus reducing unphysical self-interactions. Furthermore, the simulation box was constructed to be sufficiently large (in particular in the *z* direction) to minimize unreal periodicity effects. Although some residual artifacts may persist due to PBCs in *x* and *y*, we tried in this way to limit their impact on the global dislocation behavior that may include nonphysical annihilation events when dislocation lines interact with their own periodic images [34,35].

**Figure 5:**
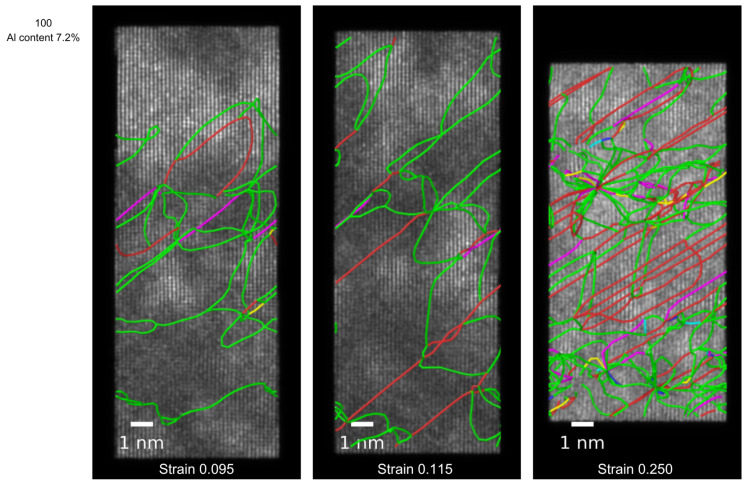
Simulated STEMs of HEA with 7.2% Al compressed perpendicular to [100], with the superposition of dislocation lines. Green lines: Shockley partial dislocations. Violet lines: Stair-rod. Yellow lines: Hirth. Red lines: unclassified (sessile) dislocations.

Other than boundary effects, the fluctuations in dislocation density may arise naturally from the dynamic balance between dislocation generation, motion, interaction, and partial annihilation. For example, dislocations can tangle or annihilate, and these effects have been measured in both simulations and experiments, particularly under conditions where dislocation interactions dominate the plastic response [36]. In our simulations, these mechanisms manifested as transient increases and decreases in the total dislocation length, especially at higher strain values where complex dislocation structures evolved. This is represented in the sequence of simulated STEMs shown in Figure 5, where we have superimposed the representation of dislocation lines for each of the three moments at which the simulated micrographs were obtained; here, we can note that, at strain values of 0.095 and even 0.115, the dislocation lines were fairly isolated from each other, but once ϵ became larger than 0.2 (micrograph on the right of the Figure), the density of dislocation was high enough for dislocations to start interacting with each other; at this stage, the system started experiencing hardening. In the figure, it can be noted that the line dislocations are primarily Shockley partials (green lines) that move via stacking fault formation. When the partials interact, they become less mobile, which contributes to hardening. Sessile dislocations, mostly those represented by the red color, also contribute to hardening. Structure analysis algorithms such as DXA or CNA are used to classify atoms in the surroundings of dislocation either as BCC or HCP, and coincidentaly, there are experimental reports that the substitution of Cu or Co by Al promotes the appearance of BCC regions and an increase in hardness [37] .

While an integral analysis of the mechanical response of the systems and the effect of Al content and direction of compression is difficult to perform given the not always clearly defined lattice defects and the fact that dislocation dynamics is in some instances too fast to be followed in detail using the recorded trajectories, some general trends can be located based on DXA and W analysis. For the compression in the [100] orientation in the system with no aluminum (0% Al), the first dislocation appeards at approximately 9.5% strain, with an increasing presence of lamellae (classified as HCP by the DXA analysis) forming in a diagonal pattern. Shockley partial dislocations (green lines in the figures) appeared at the boundaries between the HCP lamellae and the still clearly FCC structure of the rest of the material, which has a role in the occurrence of slip planes. As the compression progressed to higher values of strain, the lamellae evolved into an almost honeycomb-like structure. Local BCC structures appeared at 6.3% strain, but they remained rare until the strain reached valued close to 8.35%, at which point they became more noticeable. At higher compressions, amorphous regions increased, while FCC structures appeared to stabilize. Nanotwins (as detected by W analysis) appeared at the range of 8 to 10% strain, growing in size at higher strain values while maintaining their orientation. At around 2.4% Al, dislocations formed first, followed by the appearance of nanotwins appearing at strains close to 9%, but with a not too defined shape. At 7.2% Al, HCP lamellae formation was disrupted by dislocations; nanotwins appeared at 8.3% strain, following a zigzag pattern instead of parallel lamellae. For 9.1% Al, structural transformations became more erratic, with FCC phases appearing and disappearing multiple times before stabilizing at 17.45% compression. Nanotwin appearance also fluctuated before becoming well defined at higher compressions. At 11.2% Al, amorphous regions became common. As mentioned before, The elastic modulus decreased as the aluminum content increased.

In the case of compression in the [110] orientation, a sudden and dense dislocation formation occurred at the plastic transition, leading to significant stacking faults. Initial FCC domains remained stable, and the presence of nanotwins was rare, and when formed, nanotwins were much thinner and sparsely distributed. BCC-like structures appeared minimally at the beginning of the plastic regime, giving way to HCP structures. The presence of aluminum lowered the elastic modulus and enhanced the ductility of the material. For 7.2% Al, nanotwins with dual, V-shape orientations appeared, leading to increased diffusion. HCP lamellae evolved diagonally to the compression axis but did not form a clear pattern. For 9.1% Al, HCP lamellae interconnected, but dislocations propagated through amorphous-like regions rather than cutting them directly. Nanotwins became prominent early in the plastic regime but diminished at higher compressions. At 11.2% Al, nanotwins appeared instantly at the elastic limit, forming alongside HCP lamellae but gradually destabilizing as the compression increased.

For the [111] orientation, dislocations were significantly longer and traversed the HCP lamellae without breaking them. The density of dislocations per unit volume was higher than in other orientations. FCC structures remained stable in the elastic regime, and amorphous structures formed while maintaining the same orientation, suggesting that deformation primarily involves plane slipping rather than phase changes. Nanotwins appeared scarsely at the onset of the plastic regime but disappeared quickly. BCC structures emerged abruptly at the transition to plasticity but remained unstable. As the aluminum content increased, HCP-like and amorphous-like formations became more frequent. At 7.2% Al, stable nanotwins appeared, but they disappeared almost entirely after the strain reached 15%. With 9.1% Al, dislocations clustered in the lower region of the simulation box, concentrating the strain. The aluminum increased the fraction of amorphous and HCP regions, creating more structural disorder. At 11.2% Al, small dislocations formed in the elastic regime but disappeared instantly, likely due to thermodynamic fluctuations. Structural diffusion increased significantly, and HCP lamellae became unstable, frequently reverting to FCC structures.

**Figure 6:**
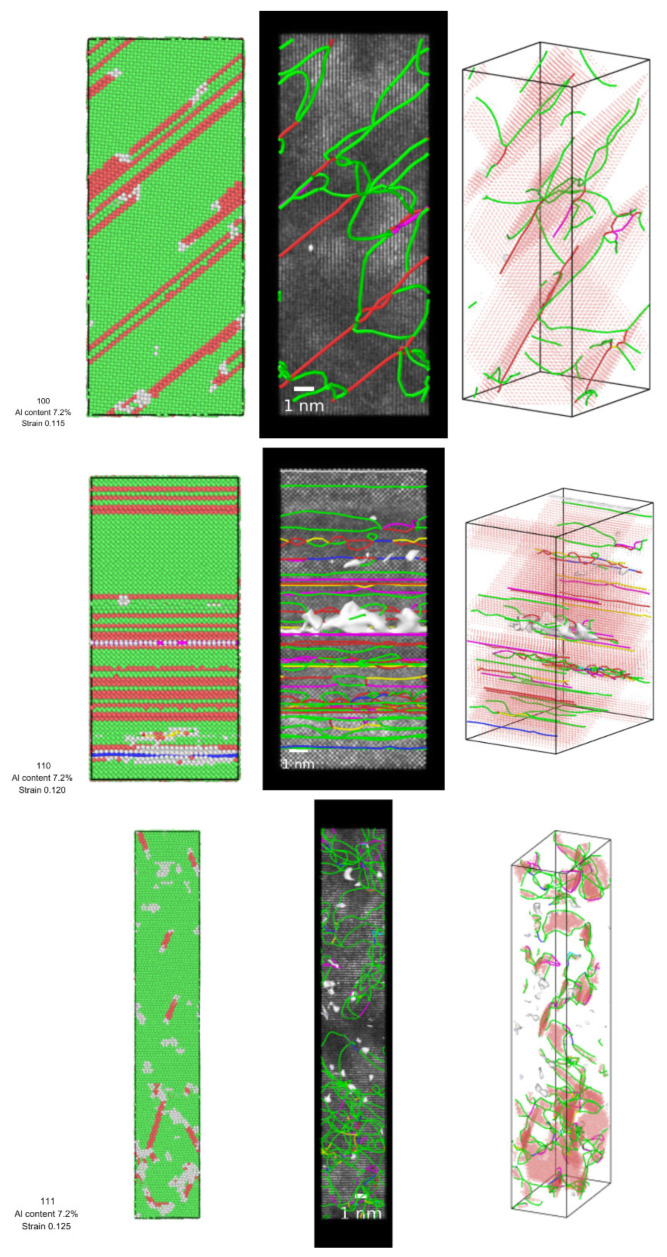
STEM simulation images with Dislocation Extraction Algorithm (DXA) analysis of FeNiCrCoAl high-entropy alloy. The images compare dislocation structures across different crystallographic orientations: [100] (first row), [110] (second row), and [111] (third row), with an aluminum content of 7.2% and varying strain levels. The left column presents atomic configurations, the middle column shows STEM simulation images with DXA overlays, and the right column displays 3D dislocation structures. Green atoms: fcc. Red atoms: hcp. White atoms: unclassified. Green lines: Shockley partial dislocations. Violet lines: Stair-rod. Yellow lines: Hirth. Red lines: unclassified (sessile) dislocations.

With the aim of aiding the interpretation of high-resolution STEMs, where the effects of thickness, local composition, and defects in the crystalline structure are intermixed, we prepared a set of composite images corresponding to simulated HAADF-STEMs for the bulk systems in the three compression directions at a strain value of approximately 0.12. We have superimposed the representation of line dislocations to the simulated micrographs. The resulting images are shown in Figure 6.

**Figure 7:**
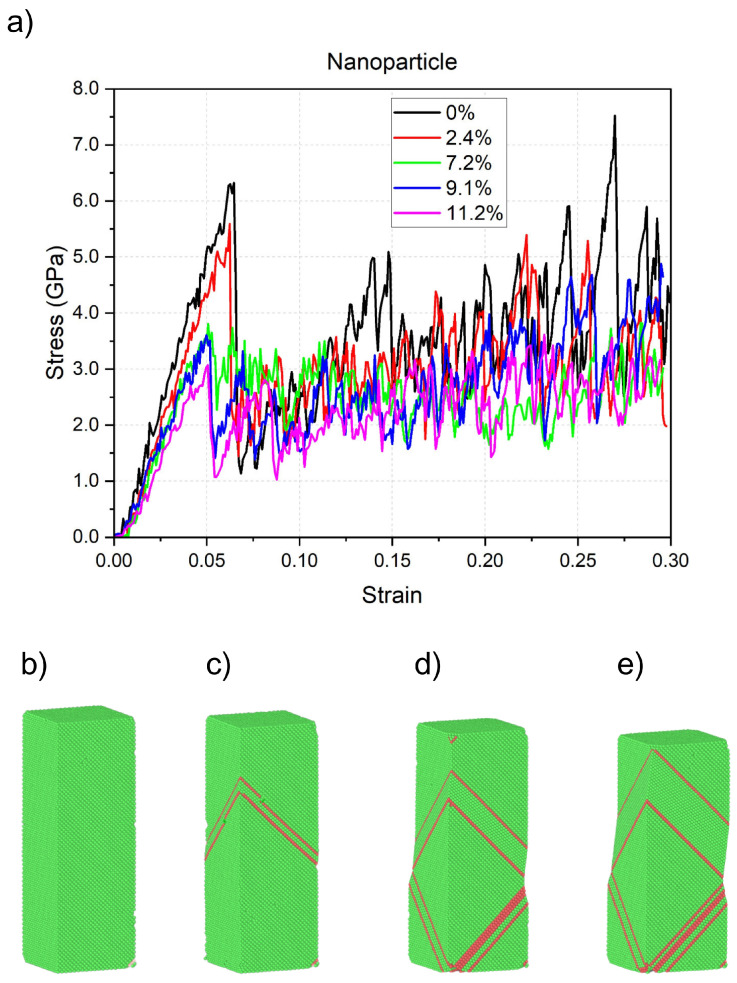
(**a**) Compressive stress vs. strain ϵ for the HEA nanoparticles at several contents of Al. (**b**–**e**) Atomistic representation of the HEA nanoparticle with 7.2 at. % Al at several values of strain ϵ: (**b**) ϵ=0.05, (**c**) ϵ=0.10, (**d**) ϵ=0.20, (**e**) ϵ=0.30.

The case of compression of nanoparticles was quite different; dislocations intermittently appeared and disappeared throughout compression, allowing for localized stability before further deformation occurred. Dislocations propagated mainly through amorphous-like structures rather than directly interacting with HCP lamellae. The FCC structures initially exhibited diverse orientations due to the free surface of the nanoparticle, preventing a fully defined structure. During plastic deformation, nanotwins formed early and persisted throughout, serving as connectors between distinct regions in the particle. At higher aluminum contents, the material showed increased diffusion of amorphous and HCP structures, suggesting greater slip activity. Structural separation phenomena were observed, with parts of the nanoparticle forming distinct regions linked by HCP lamellae. At 7.2% Al, nanotwins formed well-defined domains connected by thin HCP lamellae. For 9.1% Al, a central nanotwin appeared with minimal branching, replacing the original FCC structure. At 11.2% Al, the system underwent rapid structural reorganization, with amorphous structures playing a dominant role. In general, the presence of aluminum increased the plasticity, reduced the elastic modulus, and promoted structural transformations at lower strains. The stress/strain relations are shown in Figure 7.

In a general way, the simulations indicate that increasing the aluminum content decreases the elastic modulus and stabilizes ductility at the cost of structural coherence. The [100] orientation promotes ordered transformations, while [110] exhibits strong resistance to deformation until plasticity initiates suddenly. The [111] orientation sustains high dislocation densities, and nanoparticles show unique intermittent dislocation activity and localized stability. In Table 2, we present a summary of the general features observed for the systems considered in the study.

## 4. Conclusions

We have implemented a set of MD simulations to examine how FeNiCrCoAl high-entropy alloys, both as bulk and as a nanoparticle systems, respond to compressive loading. The results show that crystallographic orientation has a strong influence on the evolution of dislocations, stacking faults, and nanotwins; in particular, compression along the direction perpendicular to [100] generally led to progressive hardening in the plastic regime when the strain reached values close to 0.2, while orientations such as [110] exhibited a more sudden transition into plastic flow under similar strain levels. The [111] direction sustained higher dislocation densities throughout deformation, mainly of the Shockley type, underscoring the intrinsic anisotropy of FCC-type structures.

While higher Al concentrations promoted the early formation of nanotwins and increased structural reorganization, the yield strength and elastic modulus unexpectedly declined as the Al content rose. This behavior suggests that, despite aluminum’s common role as a strengthening element in many alloys, the complex lattice distortions introduced by Fe, Ni, Cr, and Co can overcome the effect of strengthening by Al.

Nanoparticles revealed different deformation patterns than bulk materials driven by the influence of free surfaces and the intermittent appearance of dislocations. Nanotwins provided plasticity in these confined geometries, sometimes linking larger regions with different crystalline configurations. At higher aluminum levels, these structural rearrangements became more pronounced, giving rise to more significant portions of HCP-like or amorphous-like domains. Despite the elevated strain rates typical of atomistic simulations and the quantitatively limited potential used in the study, the overall trends and defect dynamics obtained in the simulations may provide insights for understanding how composition, orientation, and scale control the strength and ductility of this kind of high-entropy alloy.

## Figures and Tables

**Table 1:** Elastic constants and mechanical properties of Fe, Ni, Cr, and Co. All in GPa, except for Poisson Ratio.

	Material	This Work	Literature/Experimental
$C_{11}=C_{22}=C_{33}$	Fe	189.99	153.8 [11], 154 [12]
	Ni	247.99	246.5
	Cr	198.76	410.7
	Co	264.63	260.225 [13]
$C_{12}=C_{13}=C_{23}$	Fe	159.24	121.76 [11], 122 [12]
	Ni	147.87	147.3
	Cr	140.52	106.7
	Co	191.69	160.186 [13]
$C_{44}=C_{55}=C_{66}$	Fe	76.86	76.9 [11], 77 [12]
	Ni	125.04	-
	Cr	111.73	105
	Co	142.06	110.92 [13]
Bulk Modulus	Fe	169.49	-
	Ni	181.24	-
	Cr	159.94	-
	Co	216.008	-
Shear Modulus ([100] plane)	Fe	76.86	-
	Ni	125.04	-
	Cr	111.73	-
	Co	142.06	-
Shear Modulus ([110] plane)	Fe	15.37	-
	Ni	50.05	-
	Cr	29.11	-
	Co	36.46	-
Poisson Ratio	Fe	0.455	-
	Ni	0.373	-
	Cr	0.414	-
	Co	0.42	-

**Table 2:** Main features occurring at the plastic regime considering direction of compression and Al content.

Orientation	Al Content	Main Features
[100]	0%	First dislocation at 9.5%, HCP lamellae form in a honeycomb pattern, BCC at 6.3%, nanotwins at 9.74%
	2.4%	Dislocations at 9.05%, more ductility, nanotwins at 9.25% but more diffuse
	7.2%	HCP disrupted by dislocations, nanotwins at 8.3% in a zigzag pattern
	9.1%	FCC structures appear and disappear until 17.45% compression, nanotwins fluctuate before stabilizing
	11.2%	More amorphous regions, lower structural stability, decreased elastic modulus
[110]	0%	No dislocations in elastic regime, sudden and dense dislocations at plastic transition, minimal BCC, stable FCC
	2.4%	Similar to 0% Al, but increased ductility
	7.2%	Dual orientation nanotwins, more structural diffusion
	9.1%	HCP lamellae interconnect, dislocations travel through amorphous structures, nanotwins diminish at high strain
	11.2%	Nanotwins form instantly at elastic limit but destabilize over compression
[111]	0%	High dislocation density, FCC stable in elastic regime, amorphous structures form but retain orientation
	2.4%	FCC retains stability, nanotwins weak at plastic transition, BCC appears but remains unstable
	7.2%	HCP/amorphous formations more aggressive, stable nanotwins appear but disappear after 15% compression
	9.1%	Dislocations cluster in the lower simulation box, increased amorphous and HCP fractions
	11.2%	Higher structural diffusion, unstable HCP lamellae, frequent FCC reversion
Nanoparticle	0%	Dislocations intermittently appear and disappear, allowing localized stability
	2.4%	Higher aluminum promotes greater amorphous and HCP diffusion
	7.2%	Nanotwins connect distinct regions, linked by thin HCP lamellae
	9.1%	Central nanotwin replaces original FCC, minimal branching
	11.2%	Rapid structural reorganization, amorphous structures dominate, increased plasticity

## Data Availability

The original contributions presented in this study are included in the article/Supplementary Material. Further inquiries can be directed to the corresponding author.

## Supplementary Materials

The following supporting information can be downloaded at https://www.mdpi.com/article/10.3390/nano15090652/s1. File S1: Dislocation density curves of each system. File S2: Sequence of simulated STEMs for each system obtained at five different values of strain.

## References

[1] Ma Y., He J., Zhou L., Zhang K., Gai X., Zhang X. (2022). Mechanical Properties and Impact Energy Release Characteristics of Al_0.5_ NbZrTi_1.5_ Ta_0.8_ Ce_0.85_ High-Entropy Alloy. Mater. Res. Express.

[2] Ma Y., Zhou L., Zhang K., Gai X., He J., Zhang X. (2022). Effects of Cerium Doping on the Mechanical Properties and Energy-Releasing Behavior of High-Entropy Alloys. Materials.

[3] Stepanov N., Shaysultanov D., Chernichenko R., Tikhonovsky M., Zherebtsov S. (2019). Effect of Al on Structure and Mechanical Properties of Fe-Mn-Cr-Ni-Al Non-Equiatomic High Entropy Alloys with High Fe Content. J. Alloys Compd..

[4] Chen S., Tong Y., Tseng K.K., Yeh J.W., Poplawsky J., Wen J., Gao M., Kim G., Chen W., Ren Y. (2019). Phase Transformations of HfNbTaTiZr High-Entropy Alloy at Intermediate Temperatures. Scr. Mater..

[5] Stepanov N., Shaysultanov D., Tikhonovsky M., Zherebtsov S. (2018). Structure and High Temperature Mechanical Properties of Novel Non-Equiatomic Fe-(Co, Mn)-Cr-Ni-Al-(Ti) High Entropy Alloys. Intermetallics.

[6] Zhang C., Wang H., Wang X., Tang Y.T., Yu Q., Zhu C., Xu M., Zhao S., Kou R., Wang X. (2023). Strong and Ductile Refractory High-Entropy Alloys with Super Formability. Acta Mater..

[7] Yeh J.W., Chen S.K., Lin S.J., Gan J.Y., Chin T.S., Shun T.T., Tsau C.H., Chang S.Y. (2004). Nanostructured High-Entropy Alloys with Multiple Principal Elements: Novel Alloy Design Concepts and Outcomes. Adv. Eng. Mater..

[8] Farkas D., Caro A. (2020). Model Interatomic Potentials for Fe–Ni–Cr–Co–Al High-Entropy Alloys. J. Mater. Res..

[9] Achmad T.L., Wibowo P.A., Sukma F.T. (2025). Design of High Entropy Superalloy FeNiCrCoAl Using Molecular Dynamics, Computational Thermodynamics, and Machine Learning. J. Alloys Compd..

[10] Luo L., Wu J. (2023). Molecular Dynamics Study on Nanoscale Scratch Characteristics of FeNiCrCoAl High-Entropy Alloy. Aip Adv..

[11] Vailhé C., Farkas D. (1997). Shear Faults and Dislocation Core Structures in B2 CoAl. J. Mater. Res..

[12] Mishin Y., Farkas D., Mehl M.J., Papaconstantopoulos D.A. (1999). Interatomic Potentials for Monoatomic Metals from Experimental Data and *Ab Initio* Calculations. Phys. Rev..

[13] Purja Pun G.P., Yamakov V., Mishin Y. (2015). Interatomic Potential for the Ternary Ni–Al–Co System and Application to Atomistic Modeling of the B2–L1_0_ Martensitic Transformation. Model. Simul. Mater. Sci. Eng..

[14] Thompson A.P., Aktulga H.M., Berger R., Bolintineanu D.S., Brown W.M., Crozier P.S., In ’T Veld P.J., Kohlmeyer A., Moore S.G., Nguyen T.D. (2022). LAMMPS—A Flexible Simulation Tool for Particle-Based Materials Modeling at the Atomic, Meso, and Continuum Scales. Comput. Phys. Commun..

[15] Koh S.J.A., Lee H.P. (2006). Molecular Dynamics Simulation of Size and Strain Rate Dependent Mechanical Response of FCC Metallic Nanowires. Nanotechnology.

[16] Li H., Du W., Liu Y. (2020). Molecular Dynamics Study of Tension Process of Ni-Based Superalloy. Acta Metall. Sin. (Engl. Lett.).

[17] Stukowski A. (2009). Visualization and Analysis of Atomistic Simulation Data with OVITO–the Open Visualization Tool. Model. Simul. Mater. Sci. Eng..

[18] Stukowski A., Bulatov V.V., Arsenlis A. (2012). Automated Identification and Indexing of Dislocations in Crystal Interfaces. Model. Simul. Mater. Sci. Eng..

[19] Larsen P.M., Schmidt S., Schiøtz J. (2016). Robust Structural Identification via Polyhedral Template Matching. Model. Simul. Mater. Sci. Eng..

[20] Honeycutt J.D., Andersen H.C. (1987). Molecular Dynamics Study of Melting and Freezing of Small Lennard-Jones Clusters. J. Phys. Chem..

[21] Kilymis D., Gérard C., Pizzagalli L. (2019). Ductile Deformation of Core-Shell Si-SiC Nanoparticles Controlled by Shell Thickness. Acta Mater..

[22] Olguín-Orellana G.J., De La Rosa Abad J.A., Camarada M.B., Mejía-Rosales S.J., Alzate-Morales J., Mariscal M.M. (2024). On the Mechanical Response of Graphene-Capped Copper Nanoparticles. Phys. Chem. Chem. Phys..

[23] Rangel DaCosta L., Brown H.G., Pelz P.M., Rakowski A., Barber N., O’Donovan P., McBean P., Jones L., Ciston J., Scott M. (2021). Prismatic 2.0 – Simulation Software for Scanning and High Resolution Transmission Electron Microscopy (STEM and HRTEM). Micron.

[24] Brown W.M., Wang P., Plimpton S.J., Tharrington A.N. (2011). Implementing Molecular Dynamics on Hybrid High Performance Computers – Short Range Forces. Comput. Phys. Commun..

[25] Chen W., Fu Z., Fang S., Xiao H., Zhu D. (2013). Alloying Behavior, Microstructure and Mechanical Properties in a FeNiCrCo0.3Al0.7 High Entropy Alloy. Mater. Des..

[26] Deng Y., Song H. (2024). Atomic Simulation for the Effect of Short-Range Order and Twin Boundary on Mechanical Behavior of FeNiCrCoAl High-Entropy Alloys. J. Mater. Res. Technol..

[27] Jiang J., Chen P., Qiu J., Sun W., Saikov I., Shcherbakov V., Alymov M. (2021). Microstructural Evolution and Mechanical Properties of AlxCoCrFeNi High-Entropy Alloys under Uniaxial Tension: A Molecular Dynamics Simulations Study. Mater. Today Commun..

[28] Yuan L., Tao R., Wen P., Li J., Wang S., Li D. (2023). Molecular Dynamics Simulation of Chemical Short-Range Order Strengthening in FCC FeNiCrCoAl Alloys. Phys. Condens. Matter.

[29] Li Q.J., Sheng H., Ma E. (2019). Strengthening in Multi-Principal Element Alloys with Local-Chemical-Order Roughened Dislocation Pathways. Nat. Commun..

[30] Cao P. (2022). Maximum Strength and Dislocation Patterning in Multi–Principal Element Alloys. Sci. Adv..

[31] Jian W.R., Xie Z., Xu S., Su Y., Yao X., Beyerlein I.J. (2020). Effects of Lattice Distortion and Chemical Short-Range Order on the Mechanisms of Deformation in Medium Entropy Alloy CoCrNi. Acta Mater..

[32] Yang X., Xi Y., He C., Chen H., Zhang X., Tu S. (2022). Chemical Short-Range Order Strengthening Mechanism in CoCrNi Medium-Entropy Alloy under Nanoindentation. Scr. Mater..

[33] Woodgate C.D., Marchant G.A., Pártay L.B., Staunton J.B. (2024). Structure, Short-Range Order, and Phase Stability of the AlxCrFeCoNi High-Entropy Alloy: Insights from a Perturbative, DFT-based Analysis. npj Comput. Mater..

[34] Cai W., Bulatob V.V., Chang J., Li J., Yip S. (2003). Periodic Image Effects in Dislocation Modelling. Philos. Mag..

[35] Madec R., Devincre B., Kubin L., Gladwell G.M.L., Kitagawa H., Shibutani Y. (2004). On the Use of Periodic Boundary Conditions in Dislocation Dynamics Simulations. IUTAM Symposium on Mesoscopic Dynamics of Fracture Process and Materials Strength.

[36] Shima K., Izumi S., Sakai S. (2010). Reaction Pathway Analysis for Dislocation Nucleation from a Sharp Corner in Silicon: Glide Set versus Shuffle Set. J. Appl. Phys..

[37] Li C., Li J., Zhao M., Jiang Q. (2009). Effect of Alloying Elements on Microstructure and Properties of Multiprincipal Elements High-Entropy Alloys. J. Alloys Compd..

