# Supplementary material for: Mechanical Response of FeNiCrCoAl High-Entropy Alloys at the Nanoscale: Predictions from Molecular Dynamics
Source: Nanomaterials (Basel). 2025 Apr 25;15(9):652. doi: 10.3390/nano15090652 (PMC12074405; doi:10.3390/nano15090652)
Supplement: Supplementary file 1 [file nanomaterials-15-00652-s001.zip › S1.pdf]

Dislocation Densities for Orientation [100]

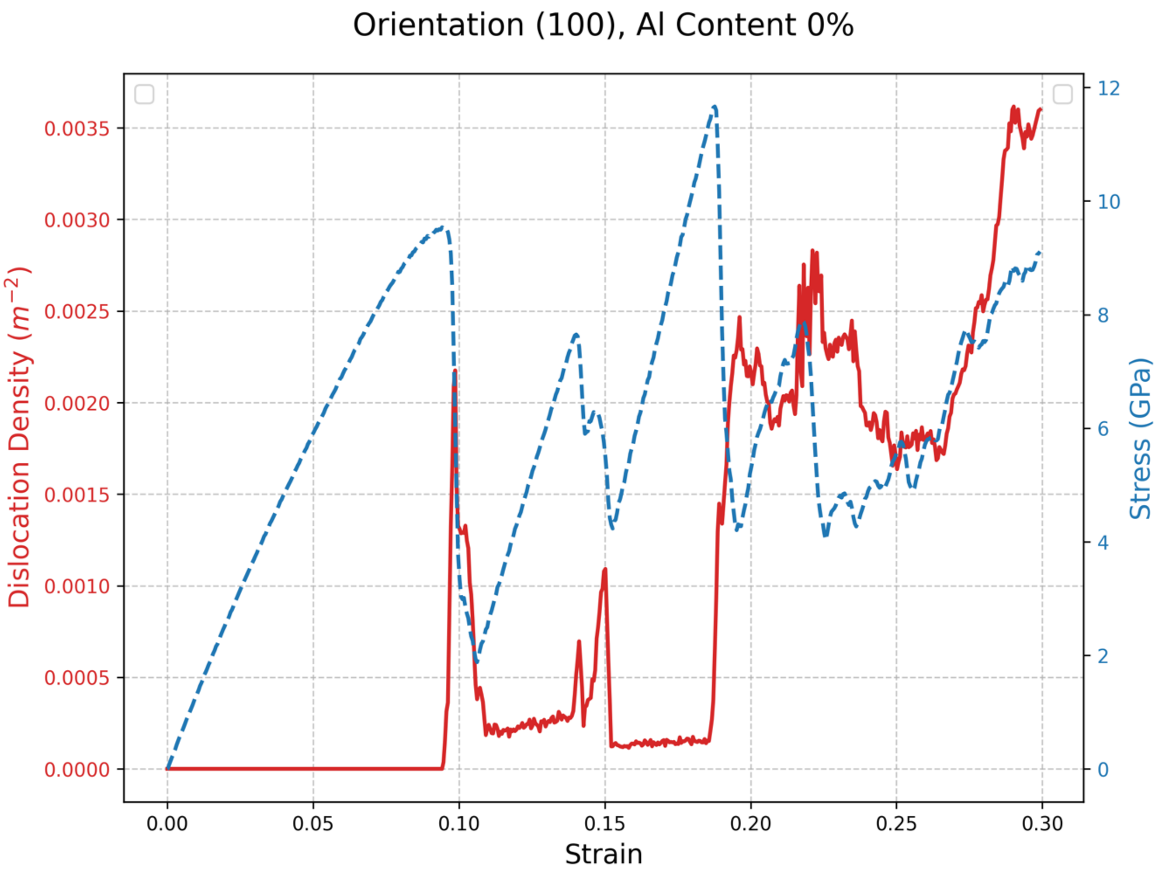

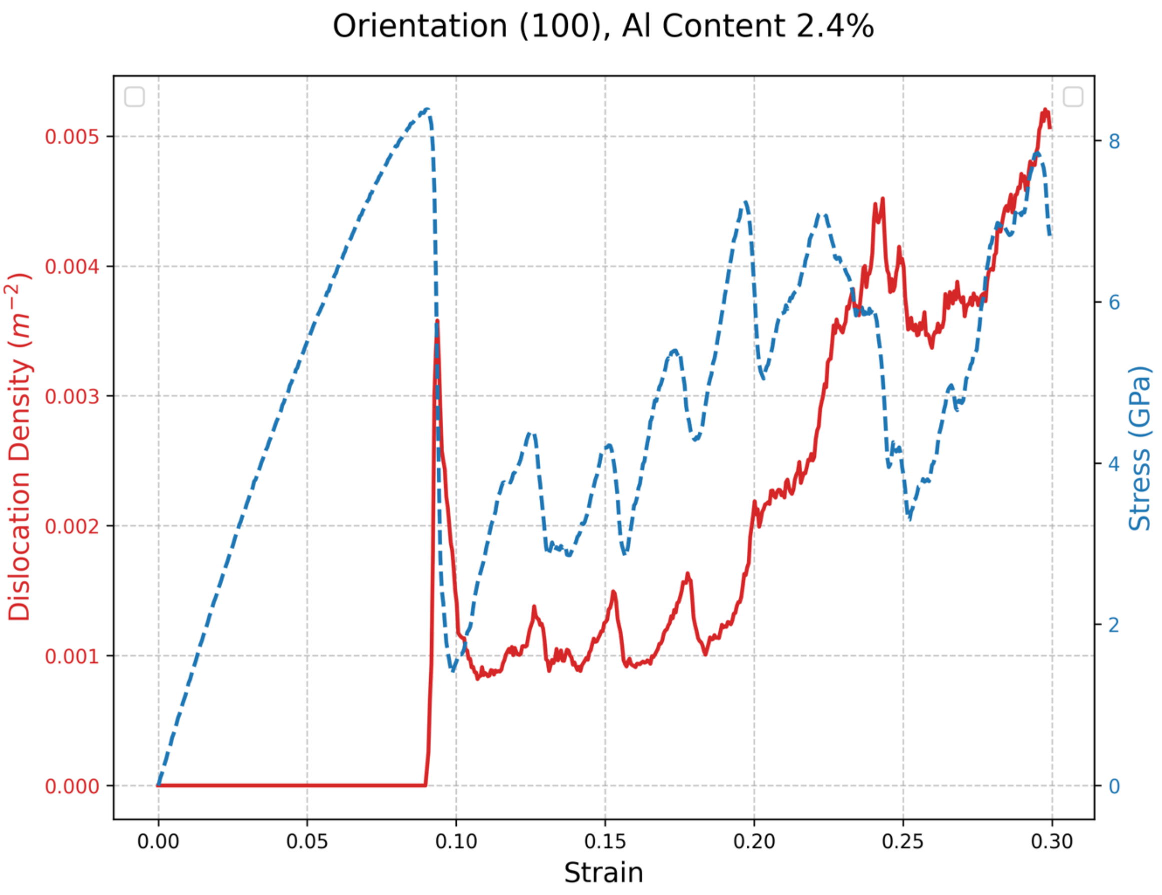

Orientation (100), Al Content 7.2%

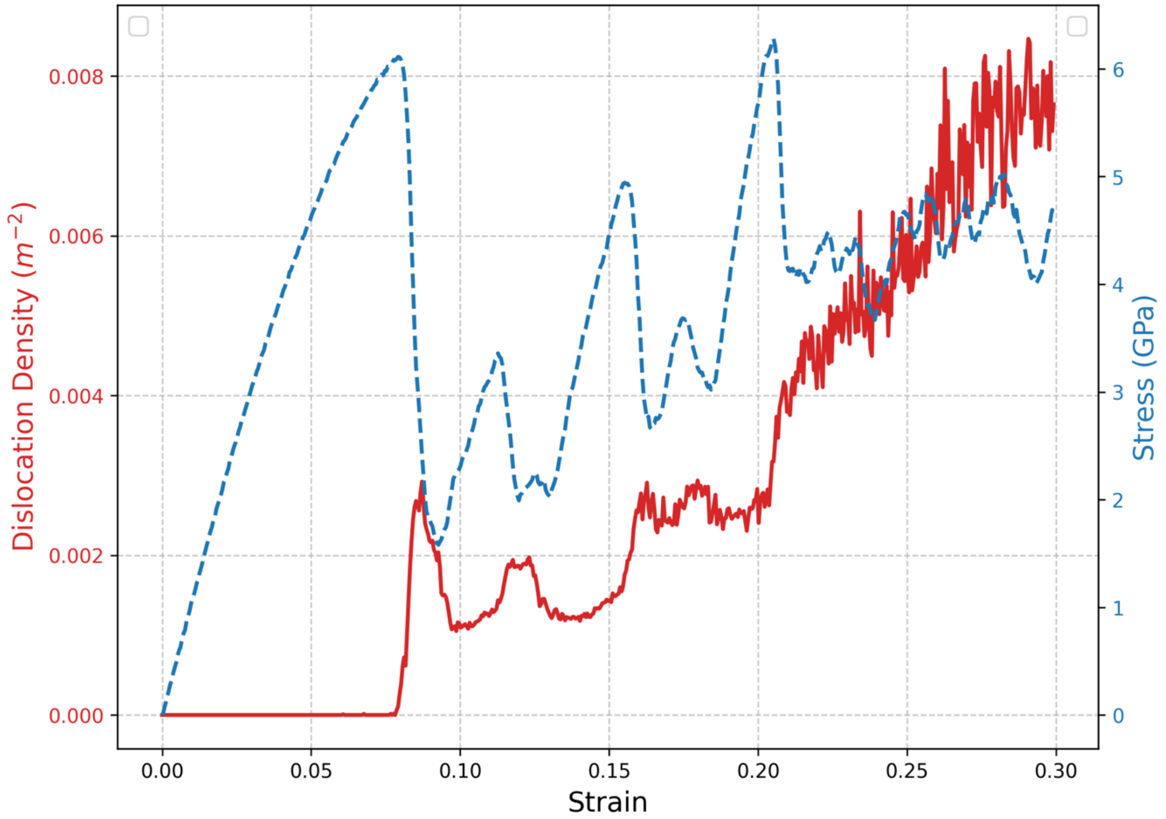

Orientation (100), Al Content 9.1%

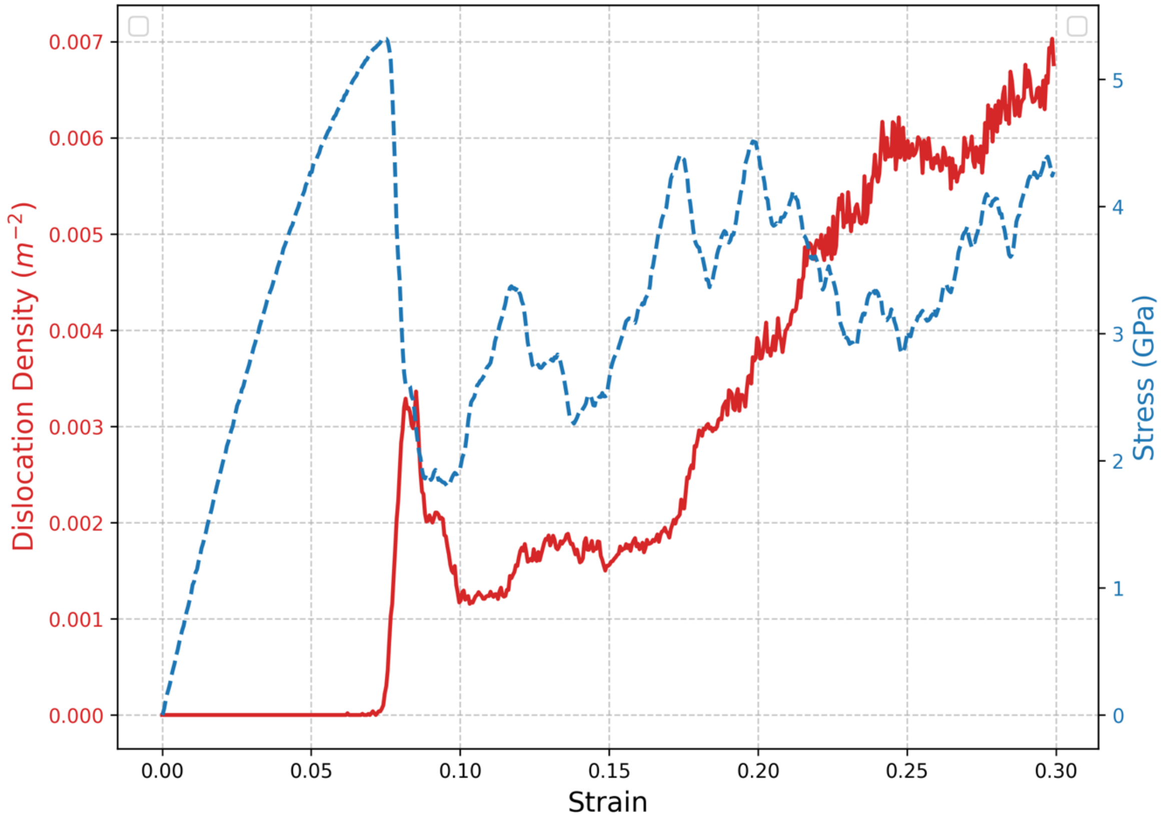

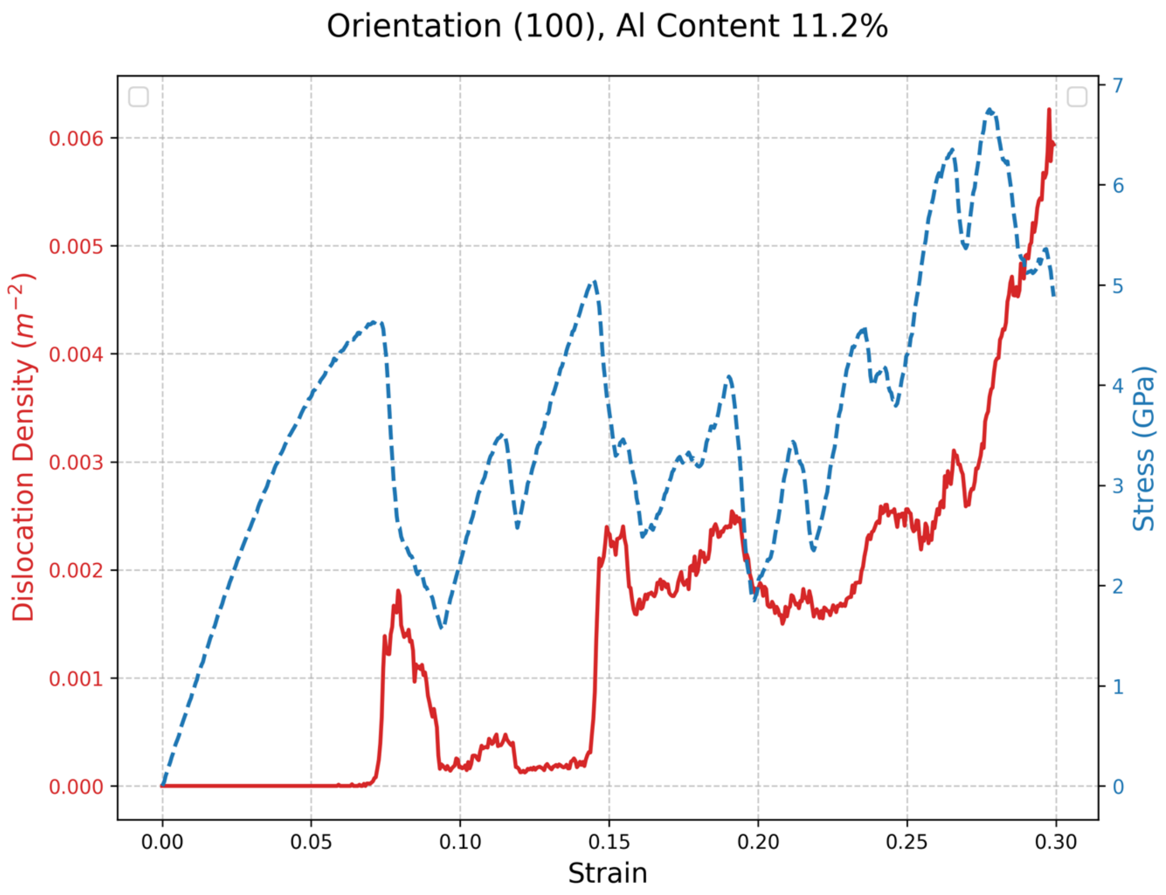
Orientation (100), Al Content 11.2%
Dislocation Density (m^{-2})
Stress (GPa)
Strain
0.000
0.001
0.002
0.003
0.004
0.005
0.006
0
1
2
3
4
5
6
7
0.00
0.05
0.10
0.15
0.20
0.25
0.30

Dislocation Densities for Orientation [110]

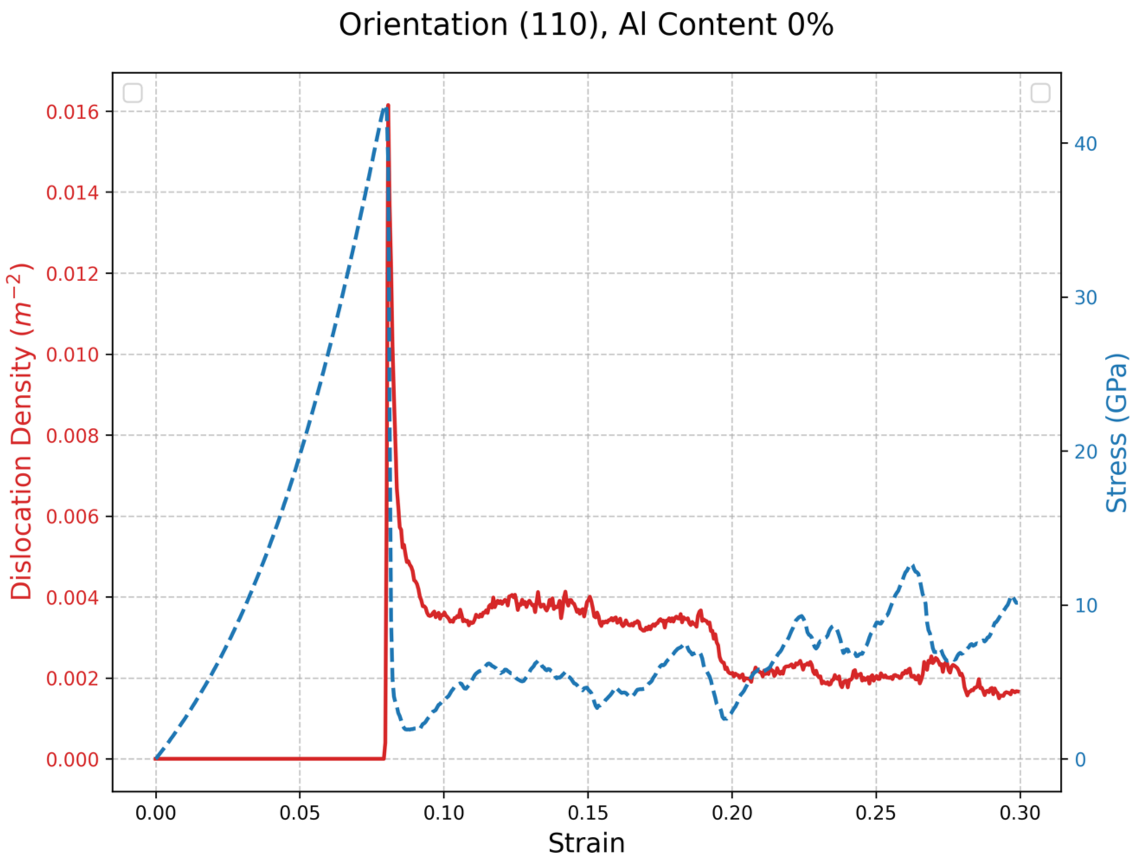

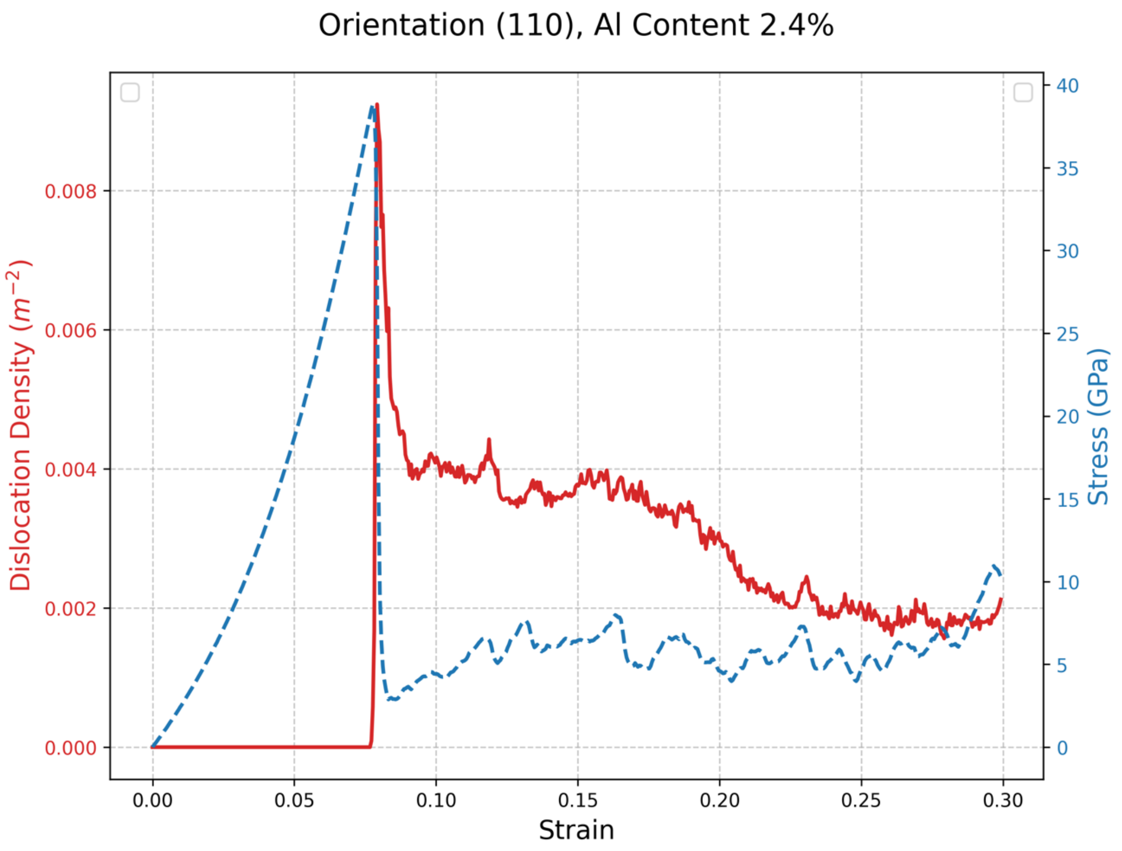

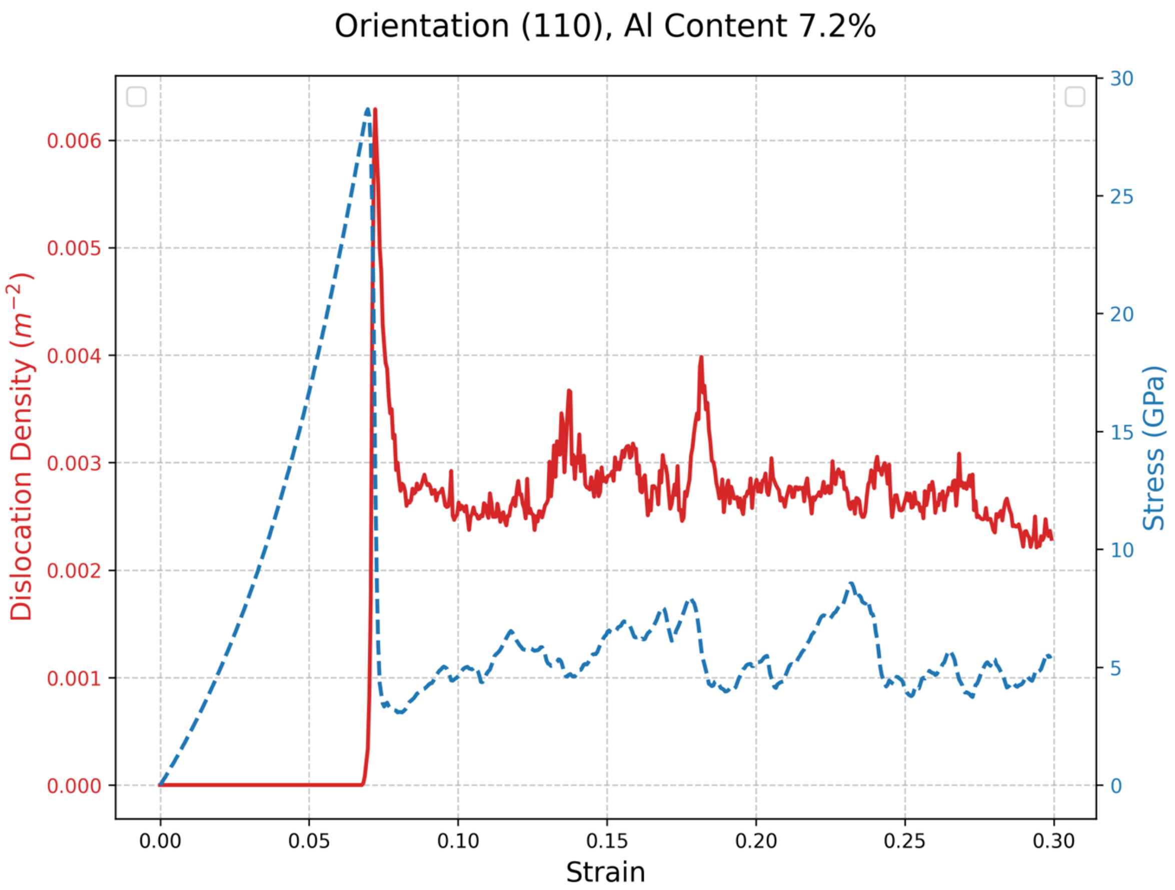

Orientation (110), Al Content 7.2%
Dislocation Density (m^{-2})
Stress (GPa)
Strain
0.000
0.001
0.002
0.003
0.004
0.005
0.006
0
5
10
15
20
25
30
0.00
0.05
0.10
0.15
0.20
0.25
0.30

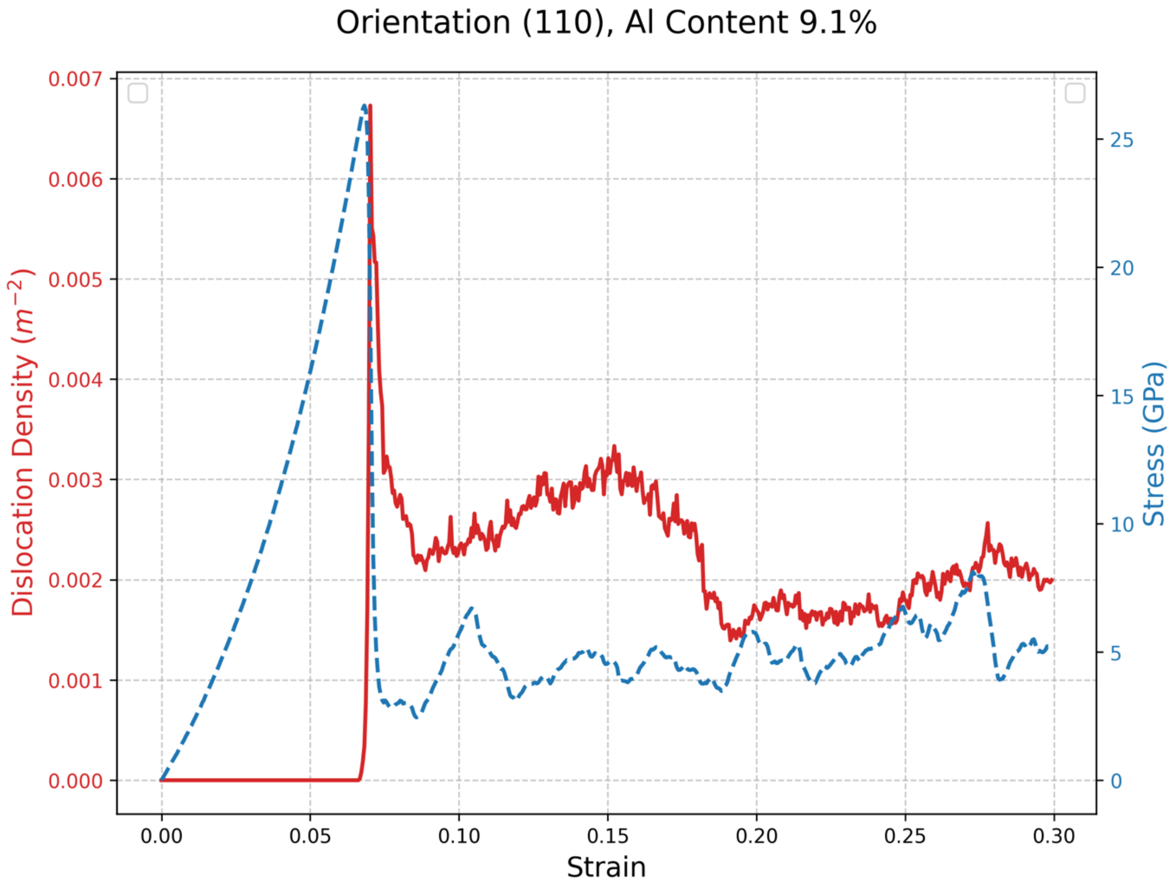

Orientation (110), Al Content 9.1%
Dislocation Density (m^{-2})
Stress (GPa)
Strain
0.000
0.001
0.002
0.003
0.004
0.005
0.006
0.007
0
5
10
15
20
25
0.00
0.05
0.10
0.15
0.20
0.25
0.30

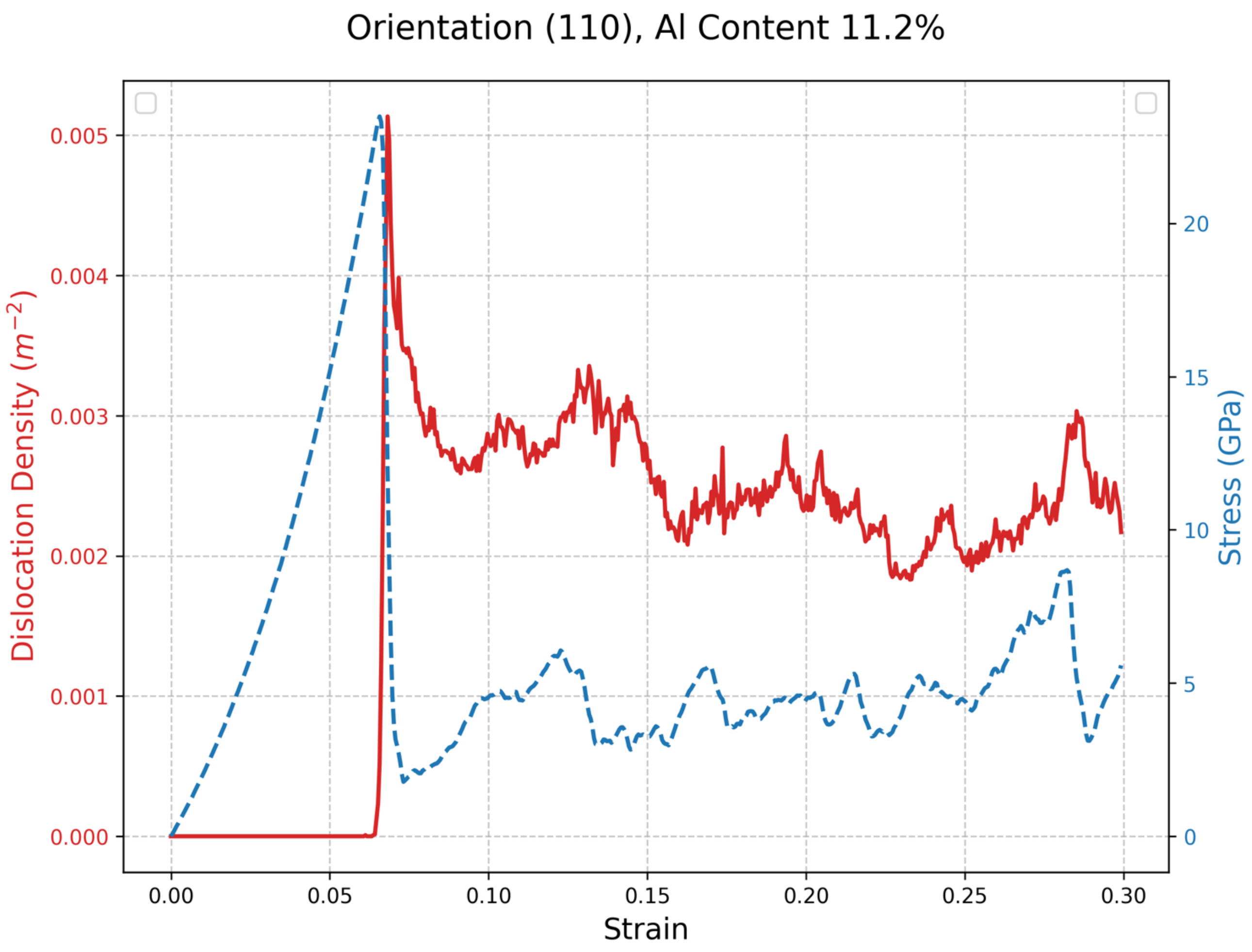

Orientation (110), Al Content 11.2%
Dislocation Density (m^{-2})
Stress (GPa)
Strain
0.000
0.001
0.002
0.003
0.004
0.005
0
5
10
15
20
0.00
0.05
0.10
0.15
0.20
0.25
0.30

Dislocation Densities for Orientation [111]

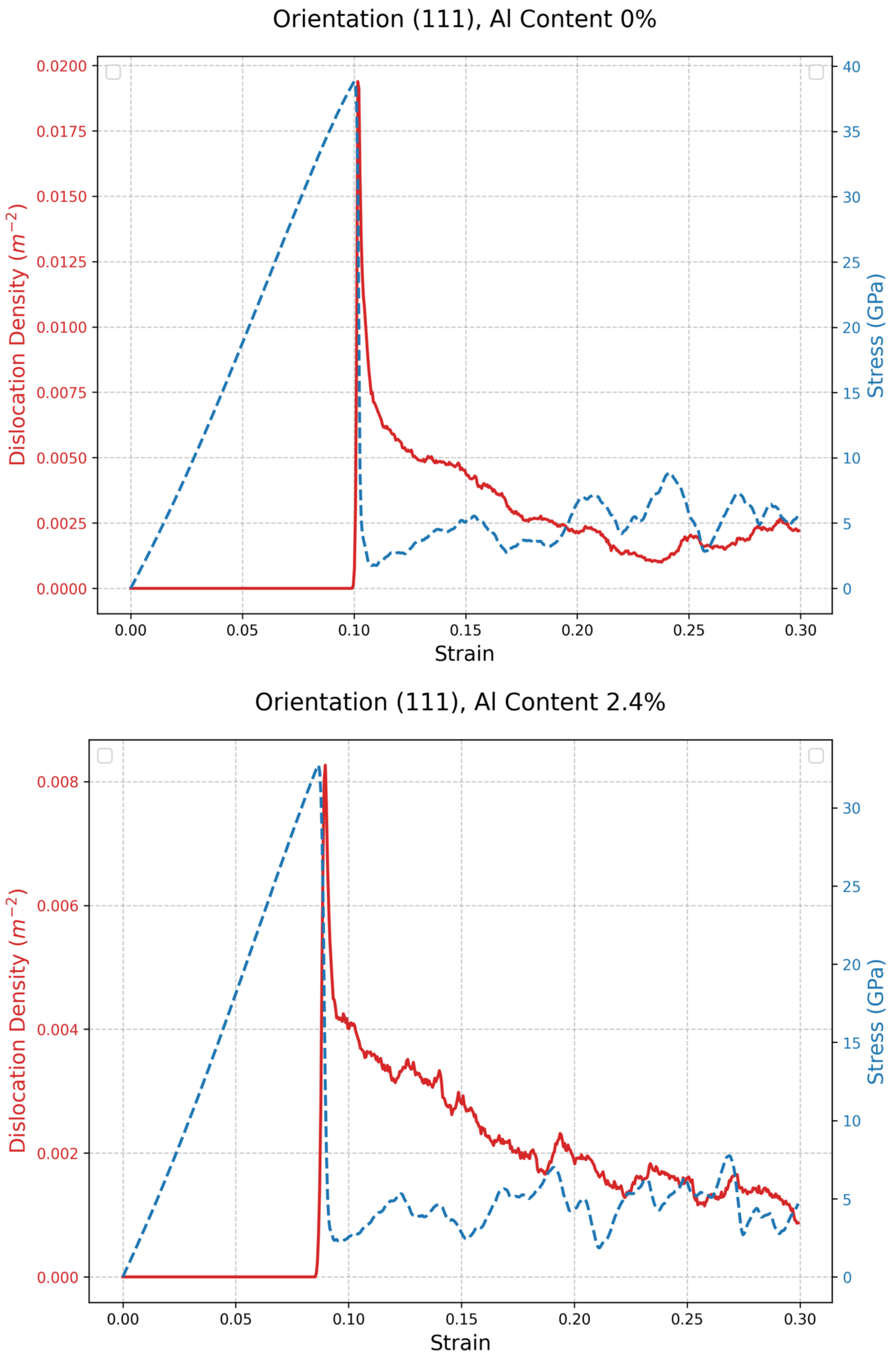

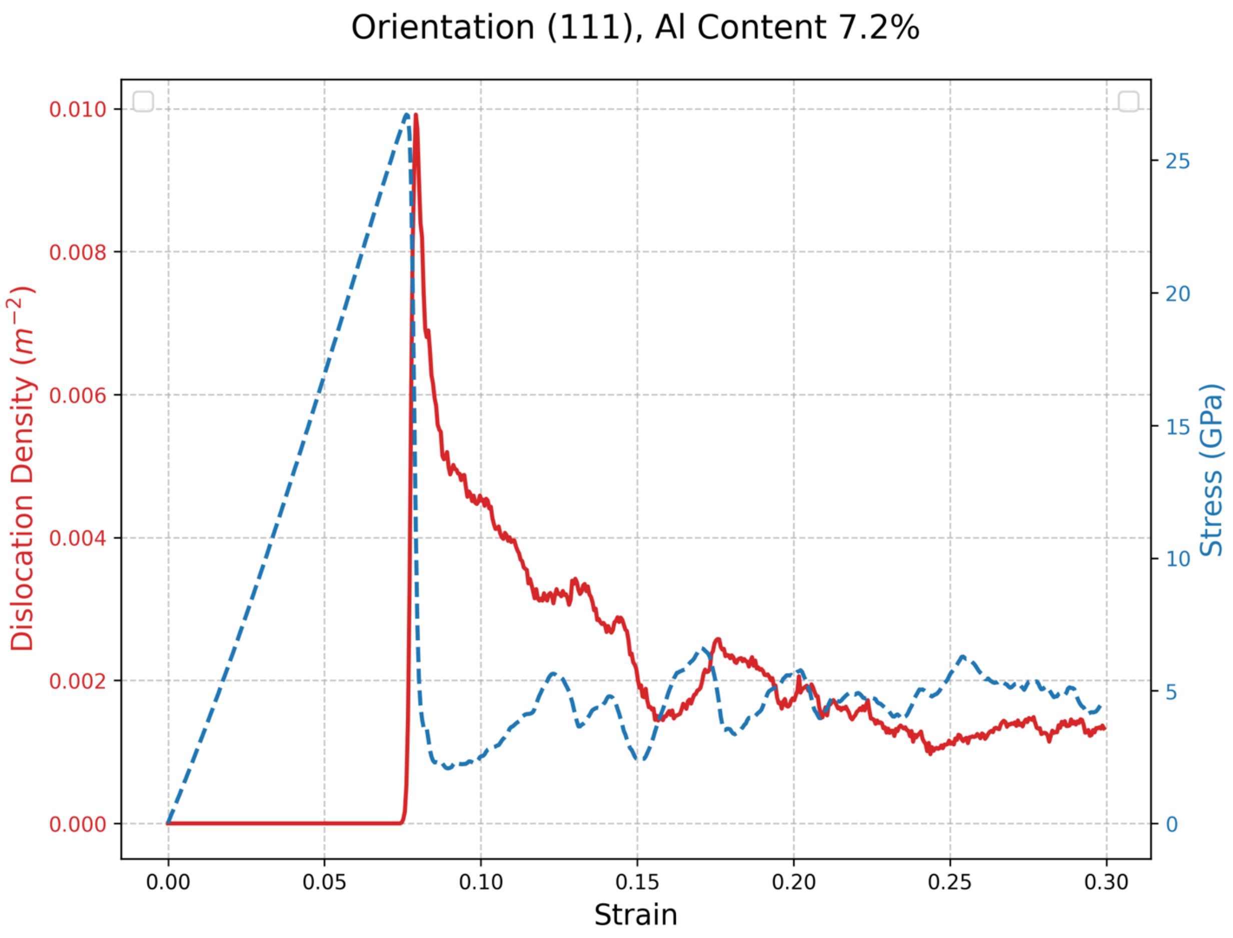

Orientation (111), Al Content 7.2%
Dislocation Density (m^{-2})
0.000
0.002
0.004
0.006
0.008
0.010
Stress (GPa)
0
5
10
15
20
25
Strain
0.00
0.05
0.10
0.15
0.20
0.25
0.30

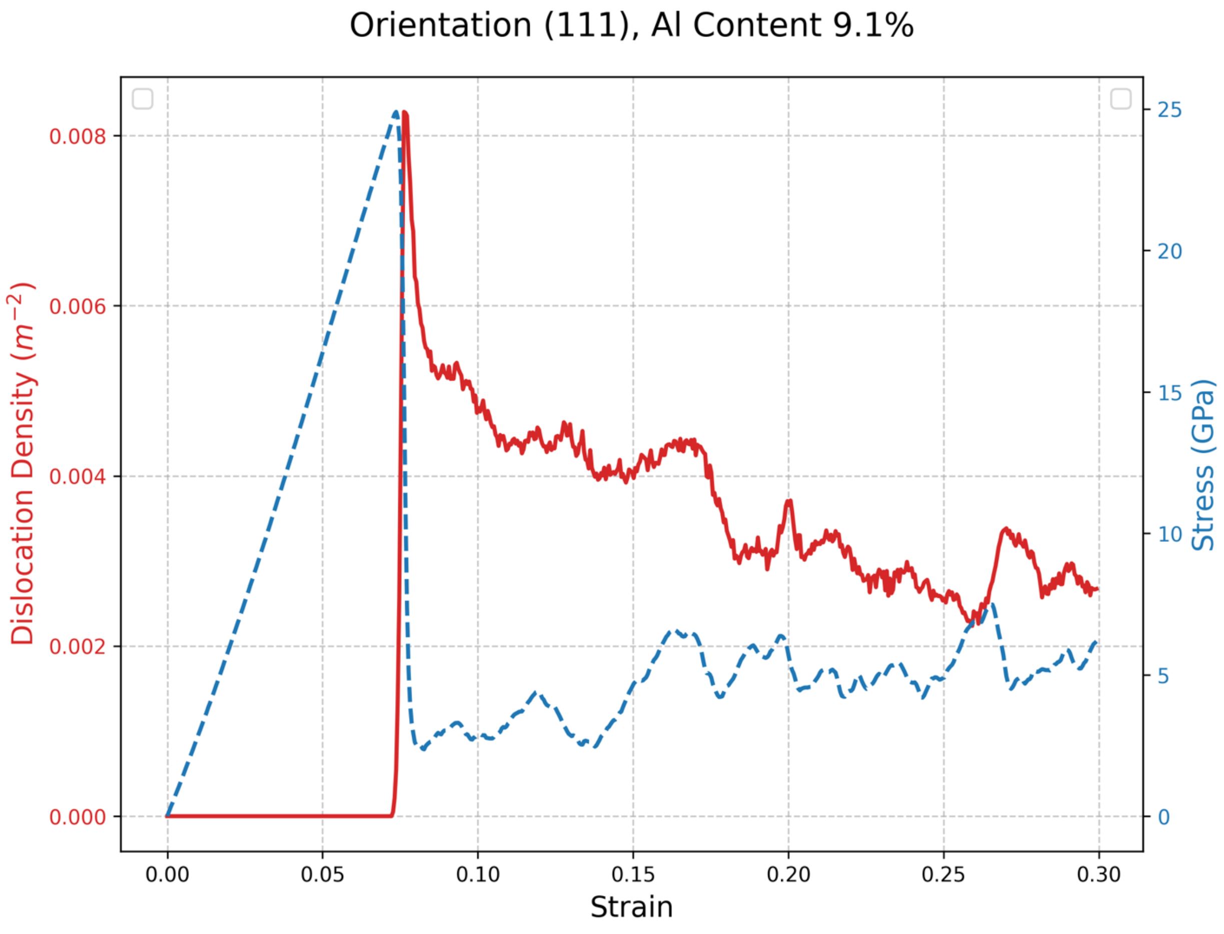

Orientation (111), Al Content 9.1%
Dislocation Density (m^{-2})
0.000
0.002
0.004
0.006
0.008
Stress (GPa)
0
5
10
15
20
25
Strain
0.00
0.05
0.10
0.15
0.20
0.25
0.30

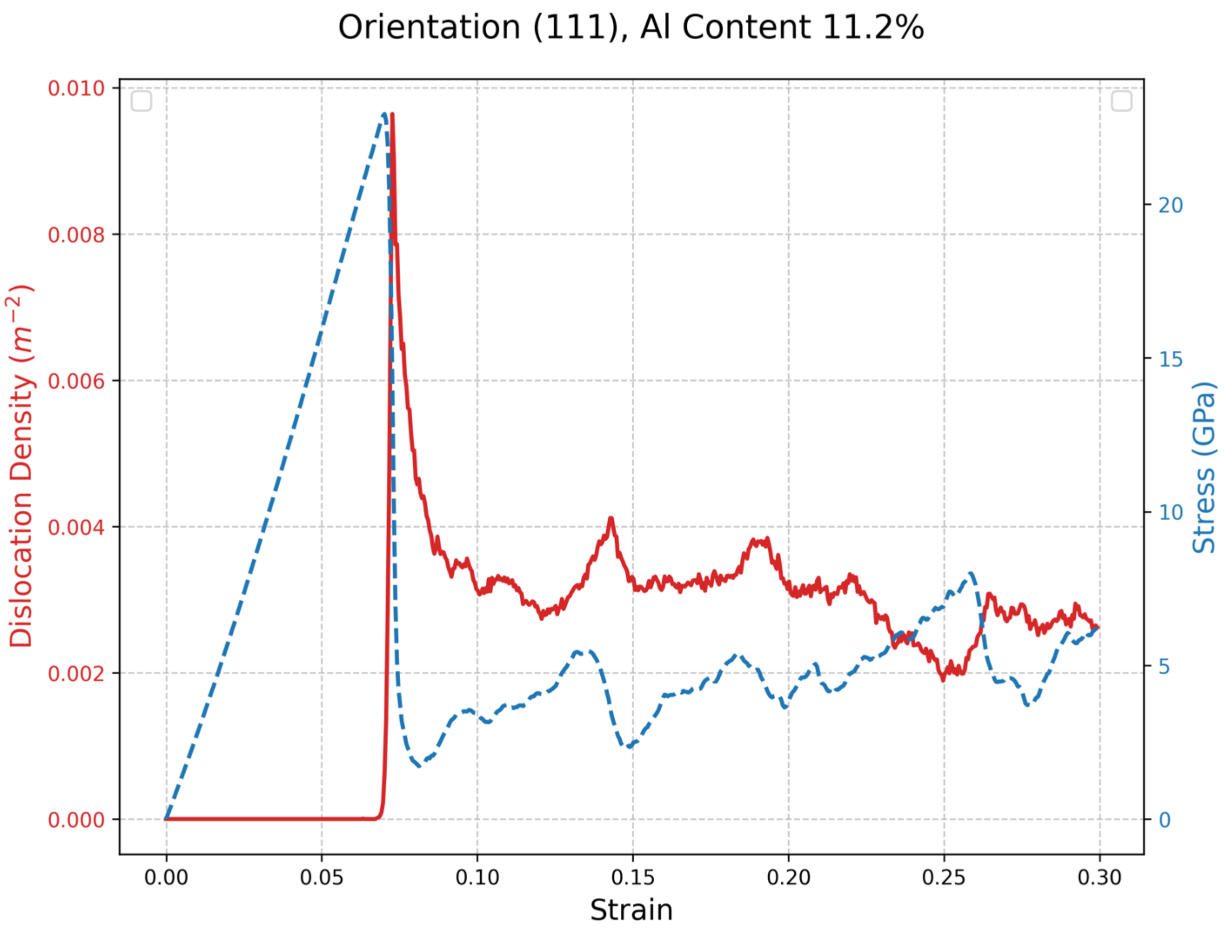
Orientation (111), Al Content 11.2%
Dislocation Density (m^{-2})
Stress (GPa)
Strain
0.000
0.002
0.004
0.006
0.008
0.010
0
5
10
15
20
0.00
0.05
0.10
0.15
0.20
0.25
0.30
