# Supplementary figures and images for: Mechanical Response of FeNiCrCoAl High-Entropy Alloys at the Nanoscale: Predictions from Molecular Dynamics
Source: Nanomaterials (Basel). 2025 Apr 25;15(9):652. doi: 10.3390/nano15090652 (PMC12074405; doi:10.3390/nano15090652)

## Orientation [100]

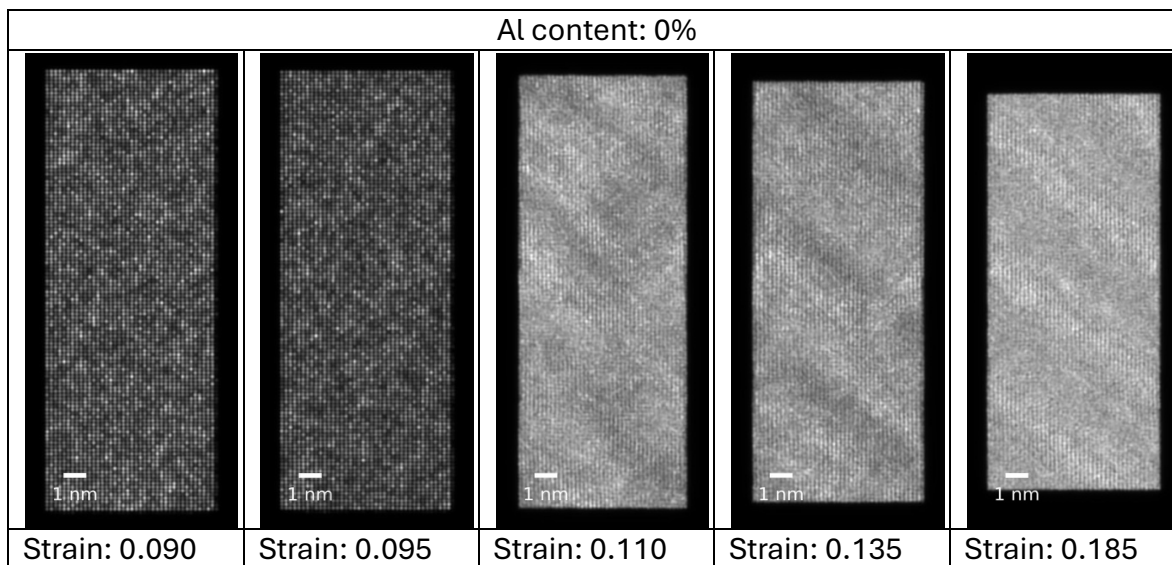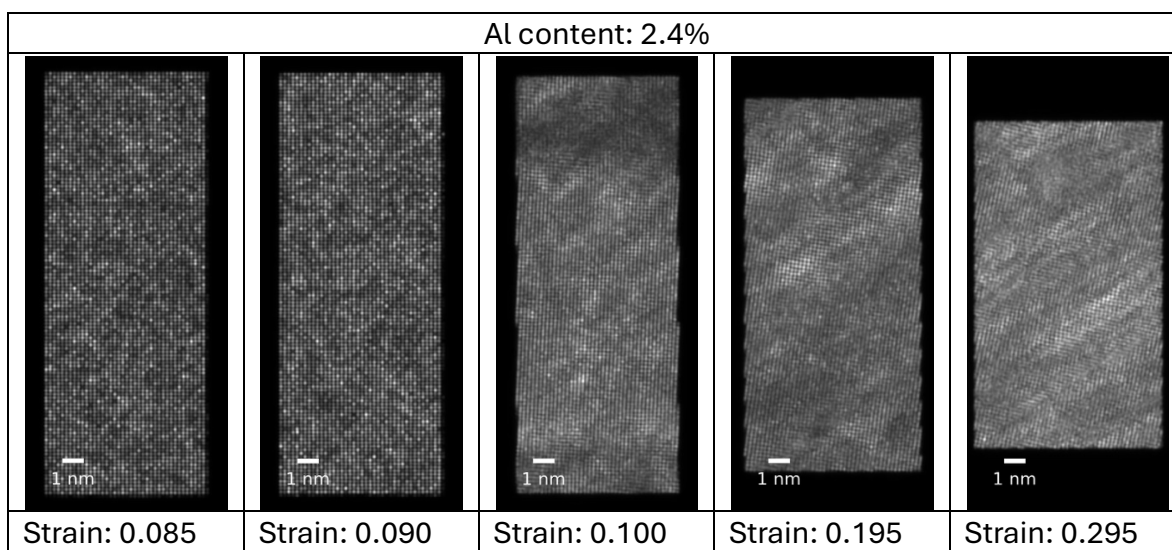

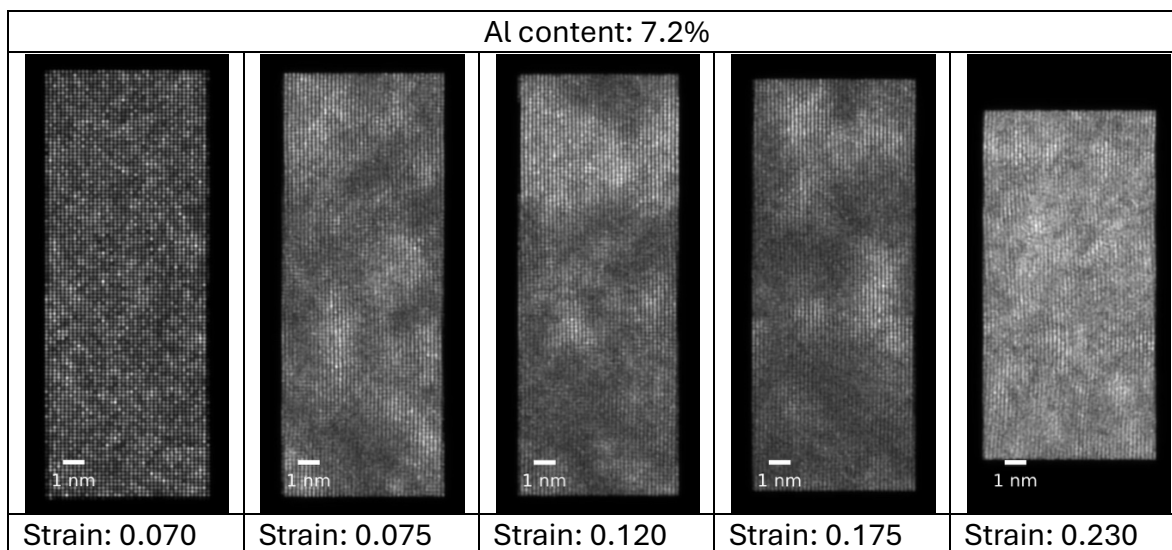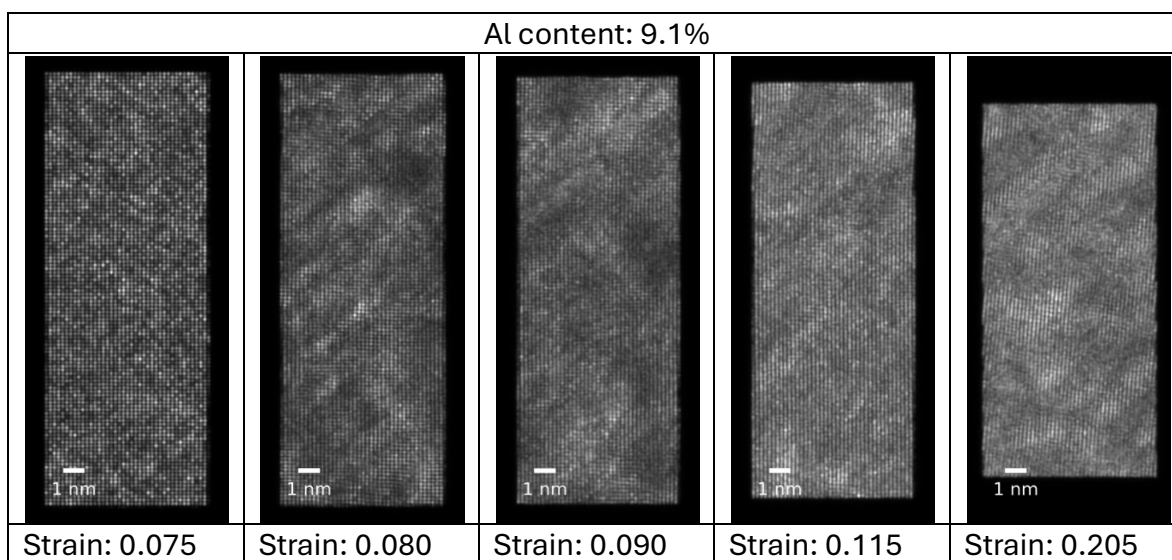

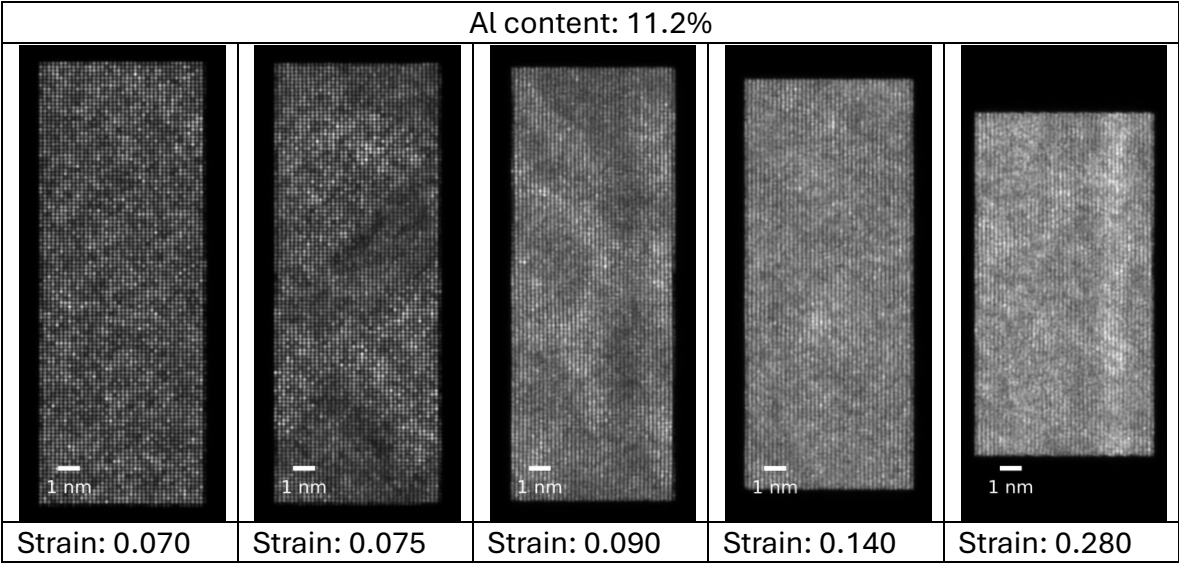

## Orientation [110]

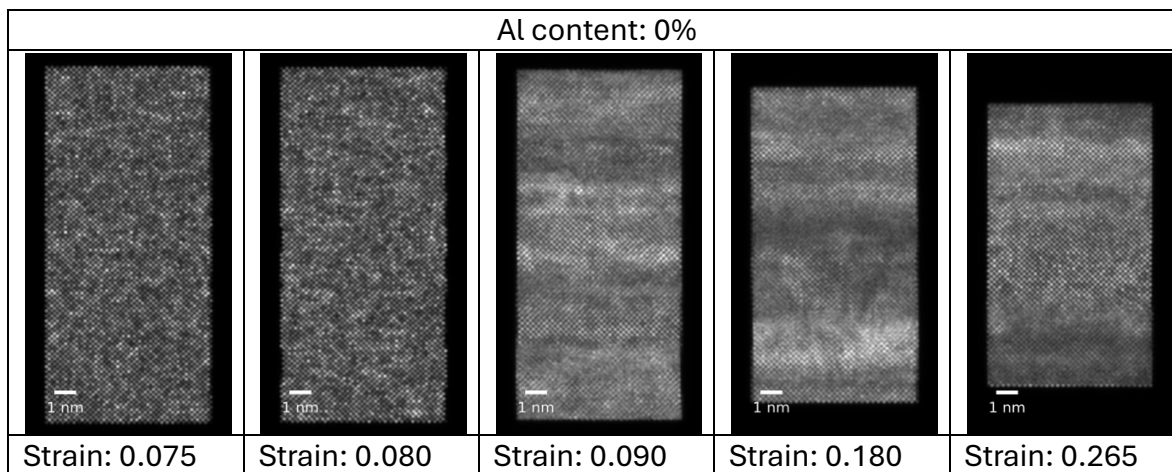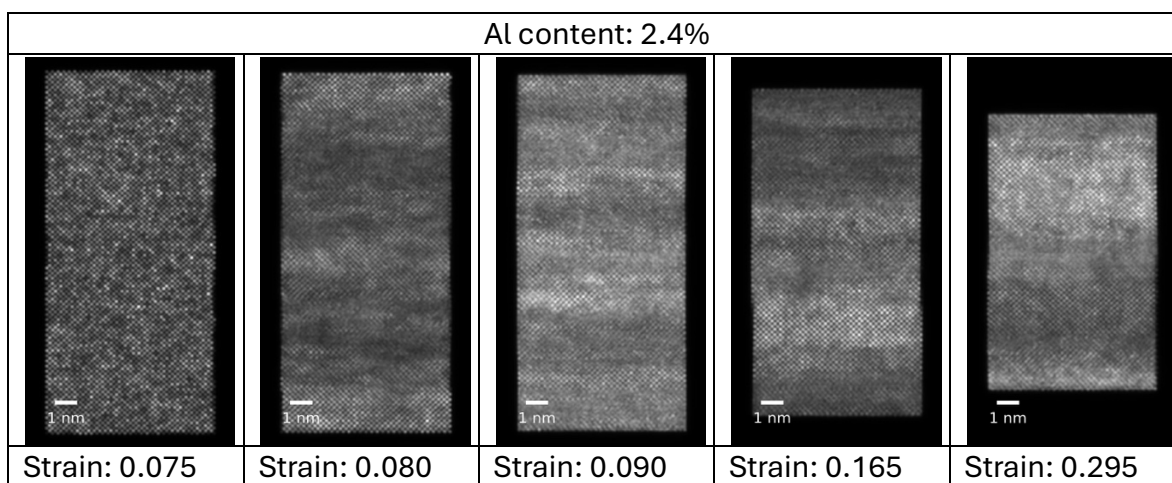

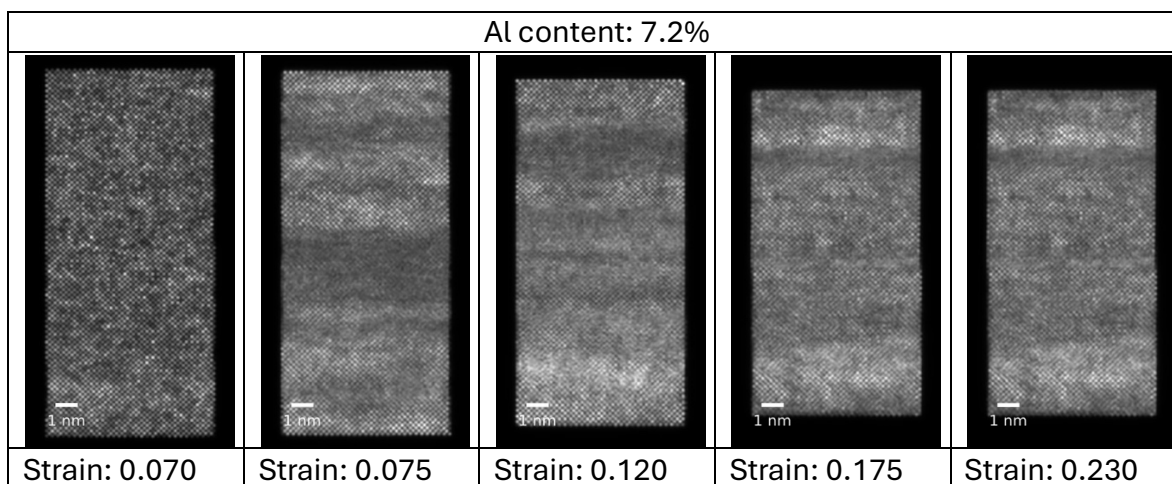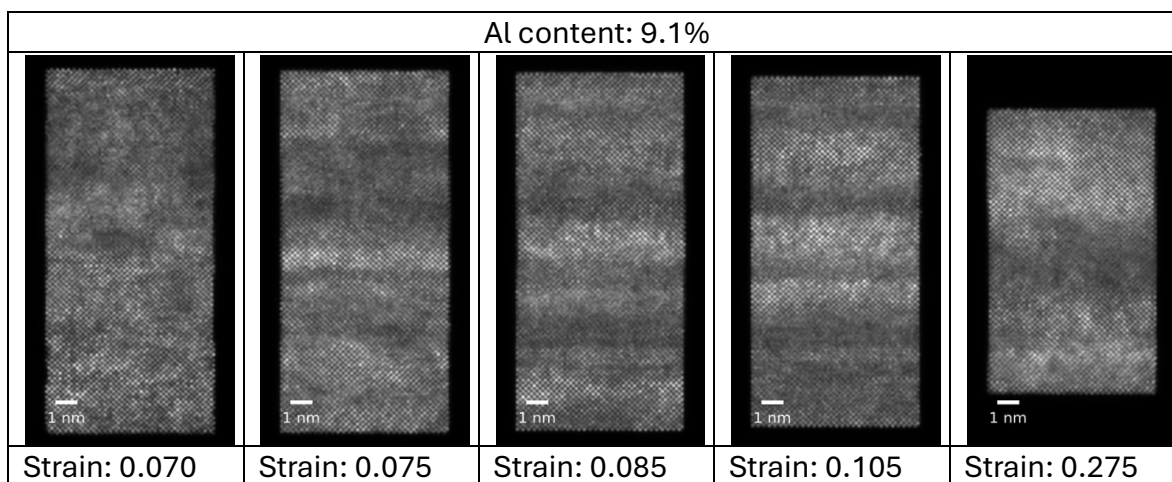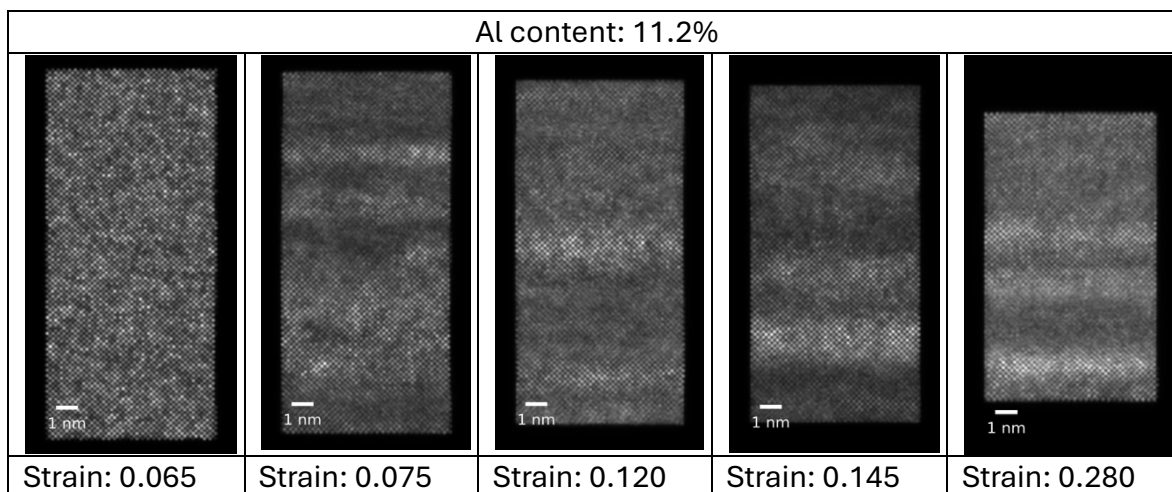

## Orientation [111]

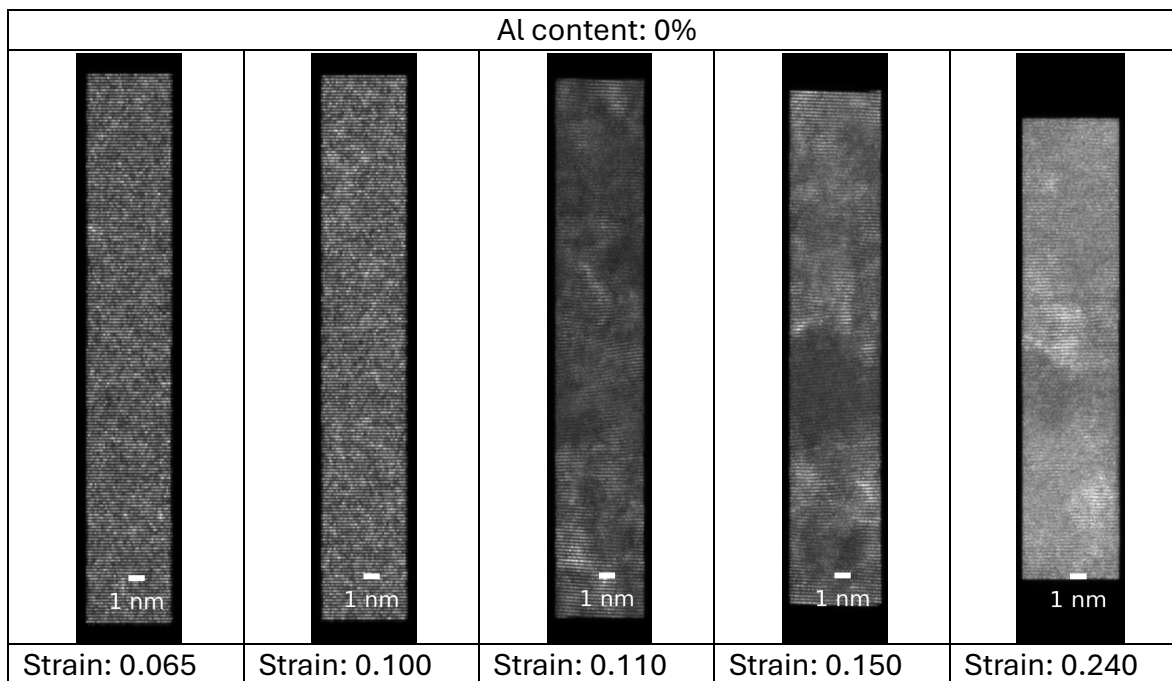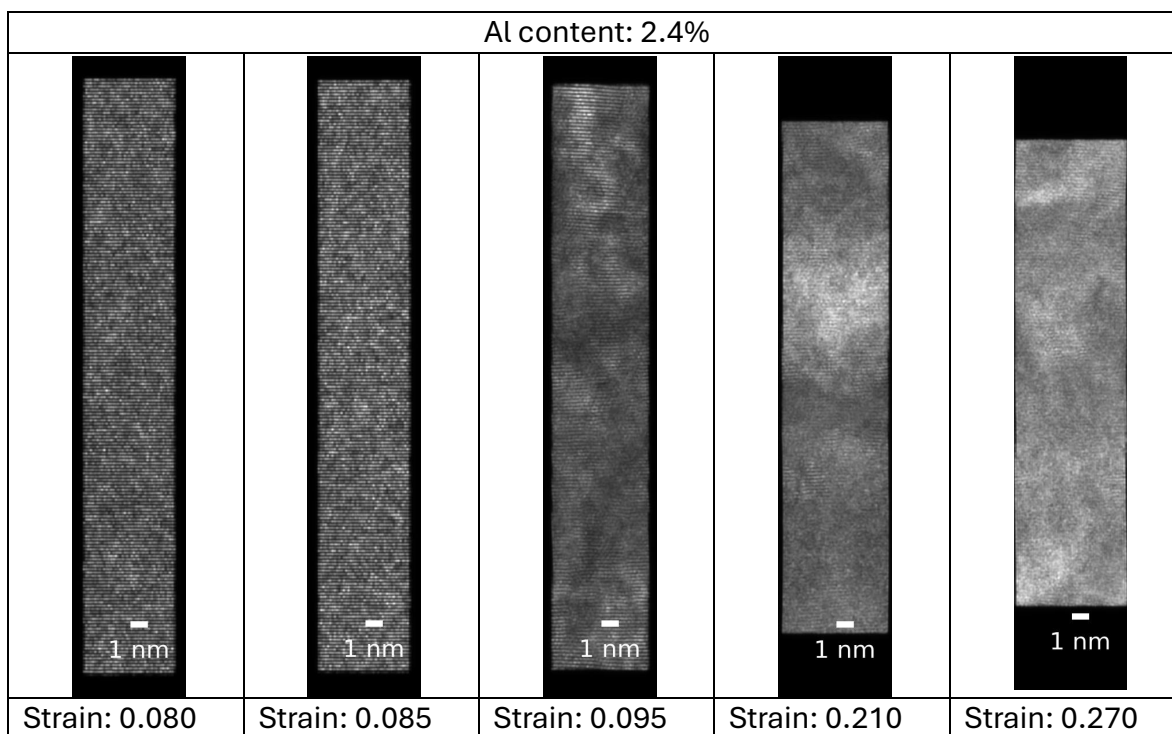

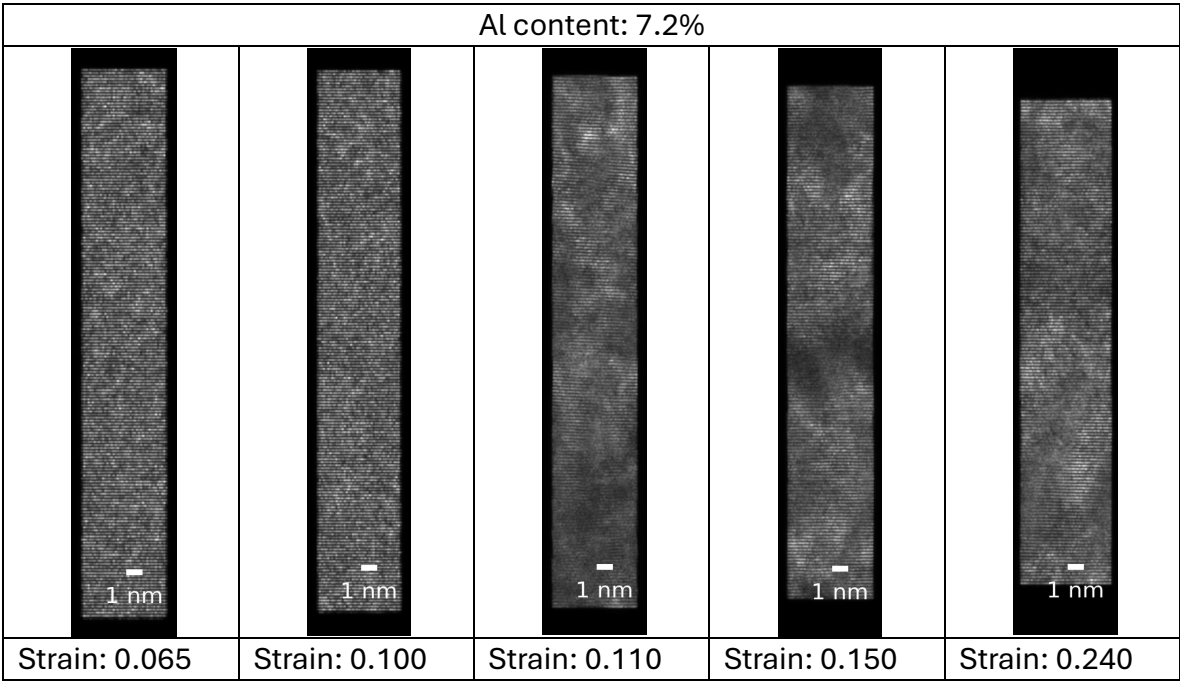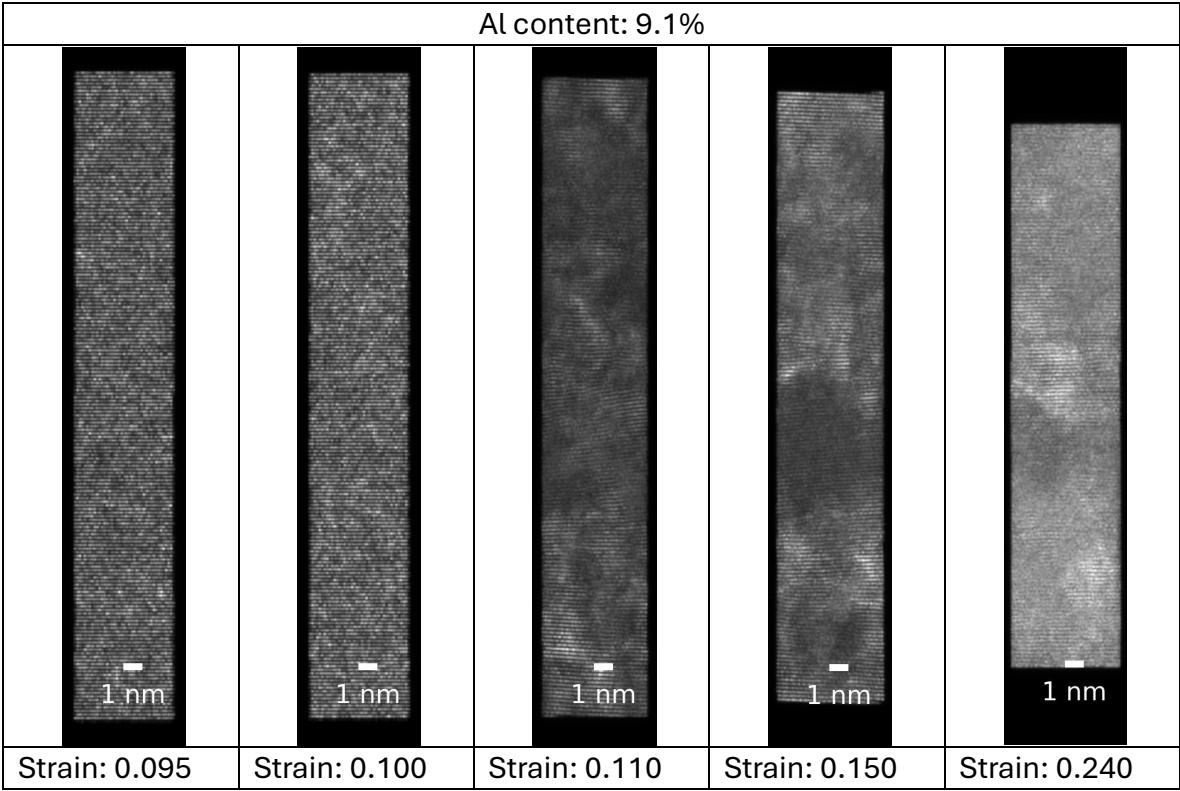

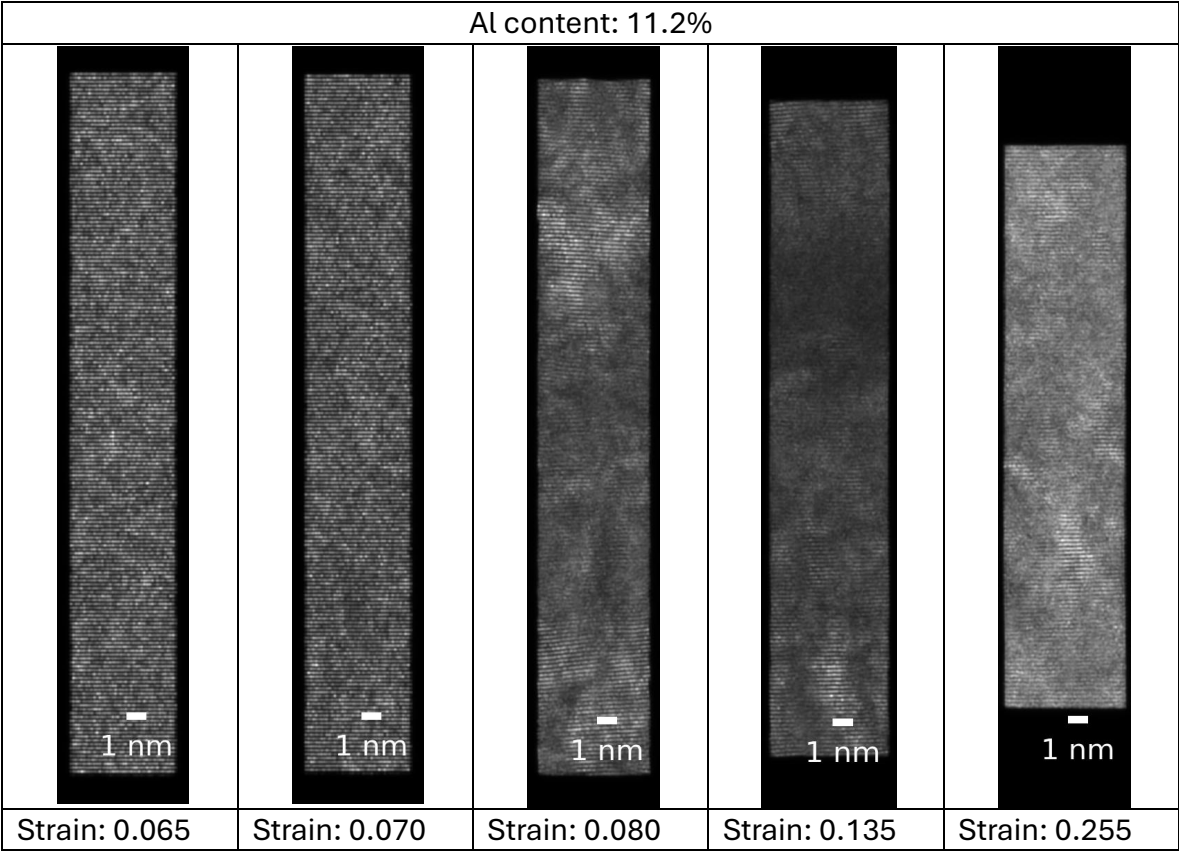

Supplement: Supplementary file 1 [file nanomaterials-15-00652-s001.zip › S2.pdf]
